# Evaluating Metagenomic Prediction of the Metaproteome in a 4.5-Year Study of a Patient with Crohn's Disease

**DOI:** 10.1128/mSystems.00337-18

**Published:** 2019-02-12

**Authors:** Robert H. Mills, Yoshiki Vázquez-Baeza, Qiyun Zhu, Lingjing Jiang, James Gaffney, Greg Humphrey, Larry Smarr, Rob Knight, David J. Gonzalez

**Affiliations:** a Department of Pharmacology, University of California, San Diego, San Diego, California, USA; b Skaggs School of Pharmacy and Pharmaceutical Sciences, University of California, San Diego, San Diego, California, USA; c Department of Pediatrics, and Department of Computer Science and Engineering, University of California, San Diego, San Diego, California, USA; d Department of Family Medicine and Public Health, University of California, San Diego, San Diego, California, USA; e Department of Computer Science and Engineering, University of California, San Diego, San Diego, California, USA; f California Institute for Telecommunications and Information Technology, University of California, San Diego, San Diego, California, USA; g Center for Microbiome Innovation, University of California, San Diego, San Diego, California, USA; University College Cork

**Keywords:** colonic Crohn's disease, inflammatory bowel disease, metagenomics, metaproteomics, microbiome, multiomics, tandem mass tags, time series, gut inflammation, proteomics

## Abstract

Although genetic approaches are the standard in microbiome analysis, proteome-level information is largely absent. This discrepancy warrants a better understanding of the relationship between gene copy number and protein abundance, as this is crucial information for inferring protein-level changes from metagenomic data. As it remains unknown how metaproteomic systems evolve during dynamic disease states, we leveraged a 4.5-year fecal time series using samples from a single patient with colonic Crohn’s disease. Utilizing multiplexed quantitative proteomics and shotgun metagenomic sequencing of eight time points in technical triplicate, we quantified over 29,000 protein groups and 110,000 genes and compared them to five protein biomarkers of disease activity. Broad-scale observations were consistent between data types, including overall clustering by principal-coordinate analysis and fluctuations in Gene Ontology terms related to Crohn’s disease. Through linear regression, we determined genes and proteins fluctuating in conjunction with inflammatory metrics. We discovered conserved taxonomic differences relevant to Crohn’s disease, including a negative association of *Faecalibacterium* and a positive association of *Escherichia* with calprotectin. Despite concordant associations of genera, the specific genes correlated with these metrics were drastically different between metagenomic and metaproteomic data sets. This resulted in the generation of unique functional interpretations dependent on the data type, with metaproteome evidence for previously investigated mechanisms of dysbiosis. An example of one such mechanism was a connection between urease enzymes, amino acid metabolism, and the local inflammation state within the patient. This proof-of-concept approach prompts further investigation of the metaproteome and its relationship with the metagenome in biologically complex systems such as the microbiome.

**IMPORTANCE** A majority of current microbiome research relies heavily on DNA analysis. However, as the field moves toward understanding the microbial functions related to healthy and disease states, it is critical to evaluate how changes in DNA relate to changes in proteins, which are functional units of the genome. This study tracked the abundance of genes and proteins as they fluctuated during various inflammatory states in a 4.5-year study of a patient with colonic Crohn’s disease. Our results indicate that despite a low level of correlation, taxonomic associations were consistent in the two data types. While there was overlap of the data types, several associations were uniquely discovered by analyzing the metaproteome component. This case study provides unique and important insights into the fundamental relationship between the genes and proteins of a single individual’s fecal microbiome associated with clinical consequences.

## Abstract (precis)

A majority of current microbiome research relies heavily on DNA analysis. However, as the field moves toward understanding the microbial functions related to healthy and disease states, it is critical to evaluate how changes in DNA relate to changes in proteins, which are functional units of the genome. This study tracked the abundance of genes and proteins as they fluctuated during various inflammatory states in a 4.5-year study of a patient with colonic Crohn’s disease. Our results indicate that despite a low level of correlation, taxonomic associations were consistent in the two data types. While there was overlap of the data types, several associations were uniquely discovered by analyzing the metaproteome component. This case study provides unique and important insights into the fundamental relationship between the genes and proteins of a single individual’s fecal microbiome associated with clinical consequences.

## INTRODUCTION

Due to the growing evidence for a connection between microbial communities and human health, exploration of the microbiome has rapidly expanded in the past decade. To date, the primary avenue for studying the microbiome has been through genomic technologies (1–3). These techniques help provide an understanding of what and how abundant the microbial constituents are and can define their associated metabolic potential. However, gene copy numbers are not representative of protein levels due to the complex systems governing when and how much of a given protein should be present (4). Further, RNA expression has been well documented to have limited correlation to protein abundance within many eukaryotes and bacteria (5). These relationships have not been thoroughly investigated in the context of the complex communities inhabiting the human gut microbiome, thus limiting the utility of DNA-based (or even RNA-based) analyses for understanding microbiome function.

Metaproteomics is an emerging technique that directly characterizes proteins from multispecies matrices. There has been over a decade of development of the field (6–9), though most studies have been limited in scope due in part to complex technical hurdles, including a lack of proteome coverage (8), sample sizes typically below 20 samples (9), limited reference database selection (10–12), and peptide assignment to proteins of similar identity (10). The introduction of new methods and instruments for use in mass spectrometry (MS) has dramatically increased the number of quantifiable peptides and proteins, allowing a greater-than-20-fold increased coverage of the metaproteome in the past few years (8, 13). Here, we leveraged tandem mass tag (TMT) technology, allowing higher throughput by combining up to 11 samples within one MS experiment, without the necessity of culturing (14). In addition, TMT workflows utilize synchronous precursor selection (SPS) and liquid chromatography-tandem mass spectrometry/triple-stage MS (LCMS^2^/MS^3^)-based quantitation workflow to increase accuracy and reduce the sparsity associated with label-free proteomics (15). This combination has enabled unprecedentedly deep characterization of proteomes at large scales (16–18). In comparison to current metagenomic technology, the metaproteome field is still limited in depth of coverage and throughput. Nevertheless, performing direct protein-level analysis through advances in MS may provide new insights into complex biological systems.

Here we utilized these technical advances to better understand the relationship between fluctuations in microbiome protein expression and fluctuations in microbiome gene content. Crohn’s disease (CD), a subtype of inflammatory bowel disease (IBD), represents a chronic autoimmune condition associated with large fluctuations in the microbiome (19–22). A study published in 2012 was the first to integrate the metagenome and metaproteome in the context of IBD (23). The results indicated that in six Crohn’s disease patients, ileal Crohn’s disease (ICD) had a unique metaproteome distinct from that associated with colonic Crohn’s disease (CCD) (23). Subsequently, a meta-analysis of human single nucleotide polymorphisms from 30,000 IBD patients corroborated the split between ICD and CCD (24). While further metaproteome studies have been conducted on the human gut microbiome of IBD (13, 25, 26), few have integrated and compared results from metagenome and metaproteome data.

A distinguishable aspect of our study is a shift from contrasting IBD cohorts and healthy subjects to exploring a time series perspective from a single patient. Previous studies investigated metaproteome stability in the context of healthy subjects (27, 28); however, those studies were limited to time periods at or below 1 year. Here, we tracked the disease activity of our patient through the abundances of several subcomponents of the immune system which form the basis of several clinical tests used to monitor IBD disease activity (29–32). These proteins include C-reactive protein (CRP), lysozyme, secretory immunoglobulin A (S-IgA), calprotectin, and lactoferrin (Table 1). Our experimental design includes one patient and eight time points, with a focus on the comparisons between metagenomic and metaproteomic data. By tracking IBD episodic dynamics through the metagenome and metaproteome, we identified a set of bacterial taxa and a set of functional groups that were found to be time-correlated with immunological biomarkers in our patient. Further, we evaluated metagenomic prediction of the metaproteome and identified unique aspects of function accessible through metaproteomics.

**TABLE 1:** Roles of immunological proteins of interest^*a*^

Protein	Role
CRP	An acute-phase response protein produced by the liver upon stimulation by IL-6, TNF-α, and IL-1-β and a common clinical marker of general inflammation (32); it is found in both human blood serum and stool
Lysozyme	A glycoside hydrolase used in the innate immune system for hydrolysis of cell walls of Gram-positive bacteria (84); measurements of lysozyme in the stool of patients with IBD have shown some correlation to disease activity in colonic IBD (84)
Secretory IgA	The most abundant antibody in the human colon; helps tightly control the relationship between commensal microbes and the host by delaying or abolishing the ability of microbes to adhere to the epithelium (49)
Calprotectin	An antimicrobial protein that sequesters manganese to prevent the growth of pathogenic microbes that require these metals (85); consisting of two subunits, S100A8 and S100A9, calprotectin is a molecule that is important to the innate immune system, constituting 40% of the cytoplasmic proteins in neutrophils; fecal calprotectin levels have been described as a stronger indicator of endoscopic activity than CRP levels, and its presence has potential for identifying endoscopic remission (29, 31, 50)
Lactoferrin	An antimicrobial glycoprotein and a major component of the secondary granules of neutrophils (50), the antimicrobial properties of lactoferrin represent the result of iron sequestration and have potential for both discriminatory and activity tests in the clinic (31, 50)

a IL-6, interleukin-6; TNF-α, tumor necrosis factor alpha.

## RESULTS

### Patient information.

The *n* = 1 patient was a nonsmoker male. He was diagnosed in 2011, at age 63, with CCD by William J. Sandborn at the University of California Health System. The inflamed region of the colon was determined, via colonoscopy and abdominal magnetic resonance imaging (MRI) analysis, to be confined to 6” to 8” of the sigmoid colon. Specifically, a 2012 colonoscopy revealed that this region had extensive diverticulosis and inflammatory focal ulceration, inflammatory pseudopolyps, and patchy friability not associated with the diverticular orifices. During the time interval covered in this work (28 December 2011 to 22 May 2016), the patient had one period of antibiotic therapy, which consisted of ciprofloxacin 500 mg administered twice daily and metronidazole 250 mg administered three times daily for 1 month starting 31 January 2012. During that period, the patient was also taking 40 mg prednisone daily. In another 4-month period from August through November 2013, the patient had simultaneous courses of mesalamine (Lialda; anti-inflammatory) and budesonide (Uceris) administered at 9 mg daily. During the reported period, the patient had episodic symptoms of rectal bleeding, abdominal cramps, bloating, and malaise. Lastly, there was no surgery performed on the patient during the time period covered by this work.

### Selection of immunological proteins of interest.

**FIG 1:**
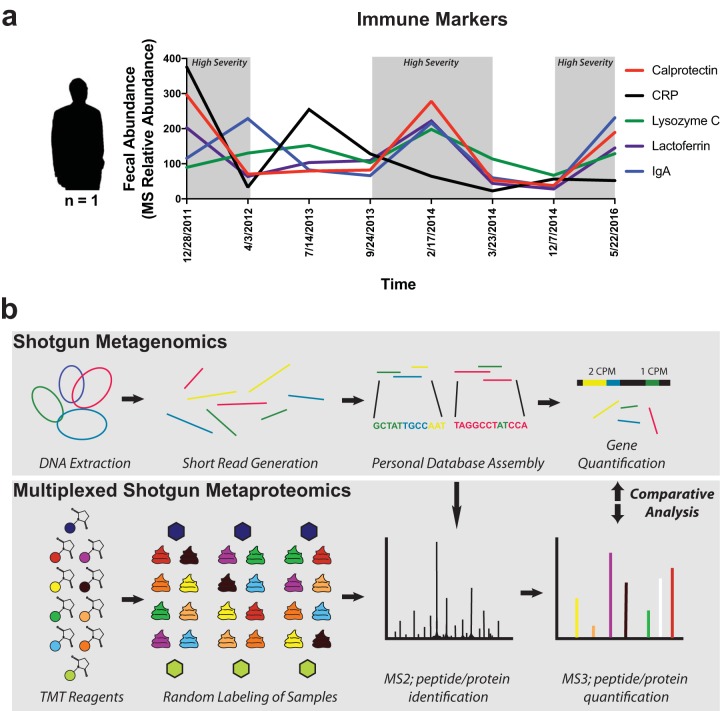
Study design. (a) Immune markers associated with samples. Mass-spectrometry-based relative abundances of fecal calprotectin, CRP, lysozyme, lactoferrin, and secretory IgA are plotted as indicated on the left *y* axis for each of the eight time points in this study. (b) Workflow schematic describing omic methods. Shotgun sequencing and metaproteomic methods were performed in parallel for the analysis of eight selected samples. Both methods were performed in technical triplicate for evaluation of technical variability. Tandem mass tag (TMT) labeling of tryptic peptides was performed for three mass spectrometry experiments. Green and dark blue hexagons represent composite samples used as controls, while other colors represent the random labeling of samples using the remaining TMT reagents. Shotgun sequencing reads were combined and assembled into a shared reference database (Personal Database Assembly) for assigning gene counts (in counts per million [cpm]) and protein abundances. Data corresponding to MS1, which was used for precursor selection, are not depicted.

The immunological proteins fecal C-reactive protein (CRP), lysozyme, S-IgA, calprotectin, and lactoferrin were selected for their unique properties and clinical applications in IBD. We observed similar expression patterns over time for calprotectin, lactoferrin, and S-IgA (Fig. 1a). Lactoferrin and S-IgA abundances were the most strongly correlated to calprotectin (Pearson *r *=* *0.96 and 0.50, respectively), which led to overlapping results in downstream analysis. Because calprotectin is more widely used for the assessment of IBD (29), we focused primarily on the relationships found with calprotectin rather than on those found with lactoferrin and S-IgA.

### Technical comparisons between –omic types and protein database methodology.

As discussed above, eight fecal samples from our patient representing a wide range of disease activity were collected over a time period from 2011 to 2016. Samples were processed in technical triplicate through the use of shotgun metagenomic sequencing and a proteomic workflow using TMT-mediated liquid chromatography triple-stage MS (LC-MS^3^) (Fig. 1b).

To address the lack of a standardized database methodology (10, 11), two different protein reference database approaches were used for analysis of LC-MS^3^ data. Our first approach utilized the shotgun metagenomic reads generated within the study to create a personalized database (pDB) containing 1.3 million protein-coding regions (23). Through alignment of our protein-coding regions to taxonomic and functional databases, the pDB provided genus-level annotations for 80% of the genes and functional annotations to KEGG orthologous (KO) groups for 15% of genes. The pDB approach was crucial for comparison between metagenomic and metaproteomic data as it provided a shared reference for gene and protein abundances. For comparison, we separately performed a two-step method (12) for searches of the MS data using a public database of gut microbial genes (the Integrated Gene Catalog [IGC]) (33). Our methods resulted in 123,806 predicted open reading frames (ORFs) from the pDB with DNA quantification and 29,370 with protein quantification (see Fig. S1a in the supplemental material). A search through both databases yielded similar numbers of peptides and proteins, with a total of 113,373 unique peptides and 72.5% of peptides shared between the pDB and the IGC database methodology (Fig. S1b). The degree of overlap in peptides was consistent with previous findings (12).

**FIG 2:**
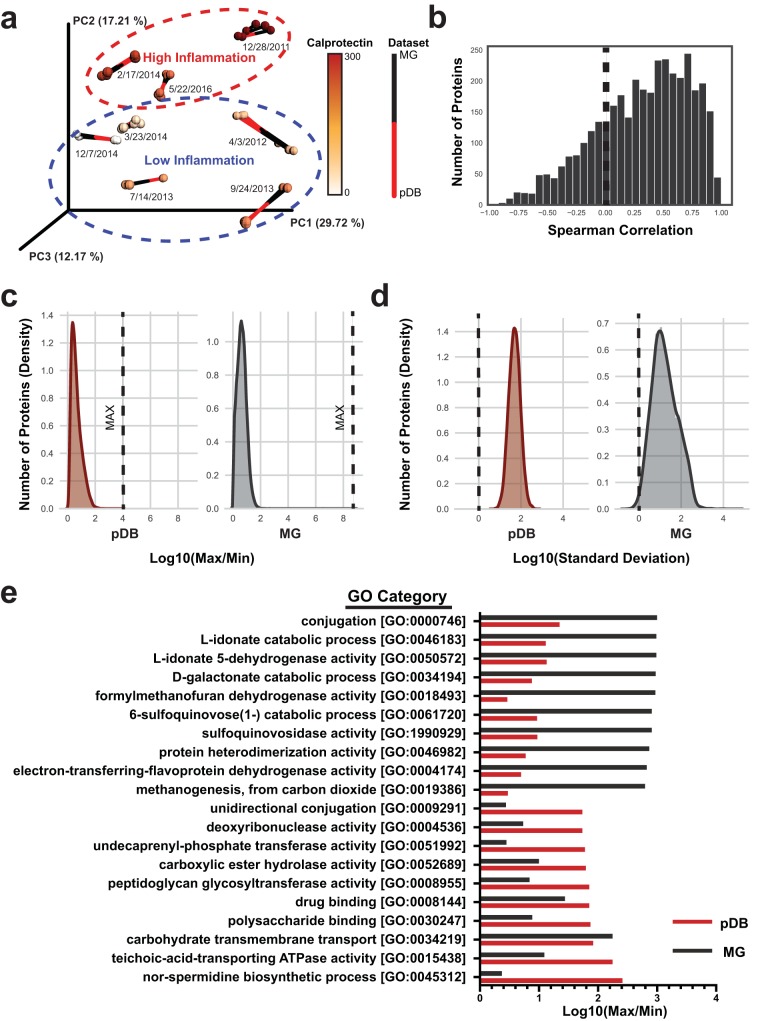
Broad-scale data type comparisons. (a) Procrustes analysis comparing clustering of the metaproteome to that of the metagenome. Bray-Curtis distance metric was used on both the metagenome and the metaproteome (only proteins common to all samples; pDB database) to assess technical and biological variability within and between data sets. Samples are colored according to calprotectin relative abundances. (b) Distribution of Spearman correlations comparing metagenomic and metaproteomic fluctuations. The *x* axis displays Spearman correlation (ρ) data, and the *y* axis displays the number of gene-protein pairs within a range of Spearman correlation values. (c) Dynamic range comparison. Histograms fitted with a Gaussian kernel density estimate are displayed at the gene and protein levels. The log 10 values representing the maximum value for each protein or gene divided by the minimum value are plotted on the *x* axis. The numbers of proteins corresponding to each maximum/minimum (Max/Min) range are plotted on the *y* axis. (d) Variability comparison. The analyses were performed as described for panel c but according to the standard deviation of each gene or protein. (e) GO categories with the largest fluctuations. Proteins and genes were summed according to their GO categories, and the maximum values were compared to the minimum values. The highest metagenomic fluctuations for each category are recorded at the top, and the highest metaproteomic fluctuations are displayed at the bottom.

10.1128/mSystems.00337-18.1 FIG S1 Technical comparisons. (a) Protein group identifications using two database methods for proteomic data and gene-level identifications against the pDB. (b) Comparisons of peptide sequence identification results from two search database methods. (c) KEGG orthologous annotation comparisons between the two search database methods for proteomics data (d) Distance comparisons of samples from cases of high and low inflammation (Fig. 2a) from Bray-Curtis PCoA data generated from the pDB average per date and MG database sums per date. (e) Spearman correlation distributions of protein and gene abundances summed at the GO level. (f) Analysis described for panel e performed using KEGG orthologous annotations. (g) Dynamic range distribution of the summed GO terms. (h) Dynamic range distribution of the summed KO terms. Download FIG S1, PDF file, 0.7 MB. Copyright © 2019 Mills et al. 2019 Mills et al. This content is distributed under the terms of the Creative Commons Attribution 4.0 International license.

Notably, a lack of sequences shared between samples is a known trait of microbiome studies (34). We observed that the TMT-based metaproteomic methods provided quantification measurements within all samples for larger percentages of proteins (52% of proteins identified from the pDB and 65% of proteins identified from the IGC) than the metagenomic techniques provided for gene quantifications (4%) (Fig. S1a). This increased overlap was likely a result of the TMT multiplexing methods, which are known to reduce sparsity in comparison to label-free MS (35). Our methods also enabled parallel quantifications of nearly 1,000 human proteins (Fig. S1a). Human protein quantification is an important advantage of metaproteomics, especially in light of recent results showing the ability of human proteins to distinguish IBD patients from controls (13). Note that the use of different databases for protein assignment can result in different functional annotations. For example, we observed that the IGC approach identified 83% more unique KEGG orthologous (KO) groups than the pDB approach (Fig. S1c). This discrepancy in peptide matching is an ongoing area of investigation in computational biology (10–12, 36).

The technical and biological variability within each data set was assessed through principal-coordinate analysis (PCoA) using the Bray-Curtis distance metric (37). To overcome the problem of the presence of structural artifacts from the missing values within TMT experiments, only the proteins common to all samples were used in this analysis. After this adjustment, a comparison between our data sets was performed using Procrustes analysis and a Mantel test (Fig. 2a). The Procrustes analysis transforms two distance matrices from corresponding samples to compare distributions. These tests showed minimal technical variability and a strong association between the two data types (Mantel test *P* < 0.001). We also observed clustering based on the presence of a state of high or low inflammation (Fig. 2a). Group differences between high- and low-inflammation states were not statistically significant, likely a result of the small number of samples analyzed. Though the data were not significant, the metaproteome showed a stronger association with the inflammation state than the metagenome (pseudo-F = 1.54 for metaproteome, pseudo-F = 1.19 for metagenome) (Fig. S1d).

To investigate the relationship between gene-level and protein-level fluctuations, the data were subsetted to the 3,598 ORFs with quantitation in both the metagenome and metaproteome. Spearman correlations between the protein and gene abundances in each of the samples were assessed. Overall, the Spearman correlations were normally distributed around ρ = 0.317 (Fig. 2b). This limited correlation highlights the added value that a metaproteomic approach can present in cases such as CD, where disease severity is associated with fluctuations in the microbiome (19). We next investigated comparisons of data types from a functional perspective by summing abundances by Gene Ontology (GO) and KO annotations and performing Spearman analyses of correlations between the genes and the protein abundances. This analysis resulted in an approximately normal distribution near ρ = 0.140 for both annotation types (Fig. S1e to f). These weak correlations might have been expected given that our approach was based on comparing DNA to protein, as even RNA abundances are often weakly correlated to protein abundance (38).

We further investigated differences in data types by comparing the distributions of dynamic ranges and standard deviations. Ratios of maxima to minima showed that both data types demonstrated a normal distribution centered around 4.4 for proteins and 11 for gene copy numbers (Fig. 2c). The maximum-to-minimum ratios reached 9,400 for proteins and 129 million for gene copy number (Fig. 2c), indicating a much greater dynamic range for the latter. These dynamic ranges may indicate the extent to which microbial genes and proteins can change over time within an individual. However, this result may be influenced by the differences in the depth of coverage, with the metagenome approaching more complete coverage than the metaproteome, and the less-abundant genes detected only by the metagenomic methods may have a greater dynamic range. The standard deviations of the genes and proteins were normally distributed but displayed differences in averages and variances (Fig. 2d). The metagenome had larger variance in the distribution of standard deviations, potentially indicating more variability within that platform (variances of 0.36 and 0.074 for the Microbial Genomes [MG] database and pDB). Still, this result may also be influenced by the differences in the depth of coverage. The values corresponding to maxima to minima for the GO and KO sums shared similar distributions between data types (Fig. S1g and h). The largest fluctuations in GO terms were greater than 100-fold for proteins and 1,000-fold for genes (Fig. 2e). Large changes were observed in categories of interest such as drug binding for proteins and methanogenesis (39) for genes. This was likely the result of the presence and then absence of two archaeal methanogens, Methanobrevibacter smithii and Methanosphaera stadtmanae (40), whose genes were, on average, 15 times more abundant at the time point of the first collection (28 December 2011) than any other sample. These results give some indication of the fundamental dynamics of genes and proteins but were surely influenced by the techniques used in the study design.

### Copy number prediction of protein abundances by functional categories.

**FIG 3:**
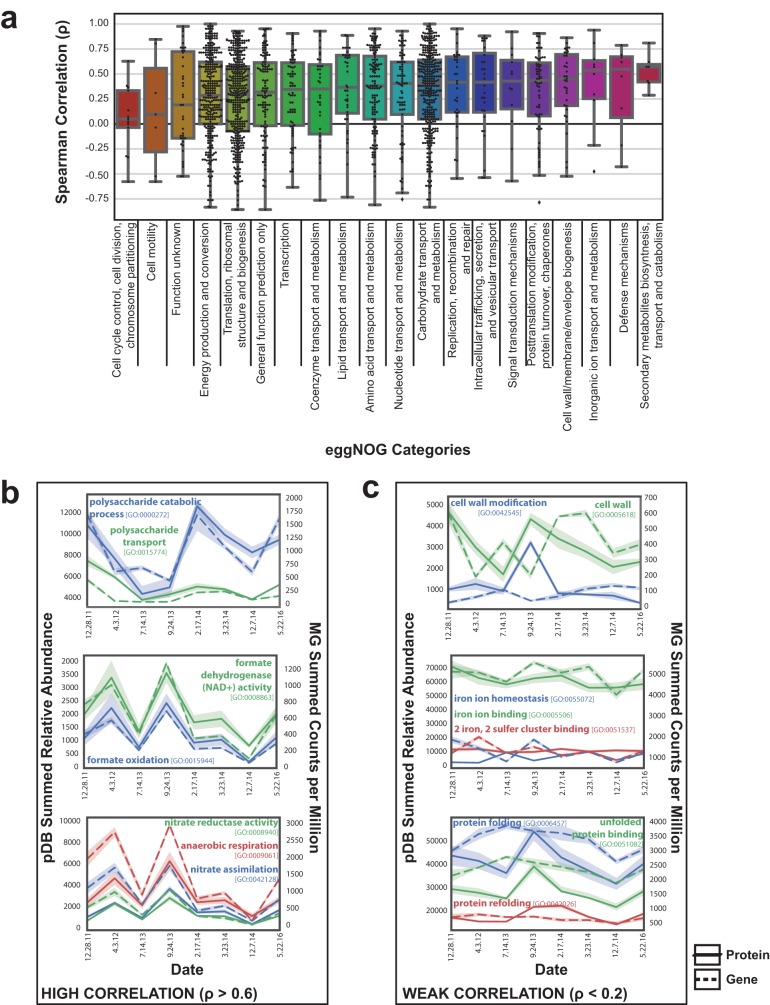
Functional categories with strong or weak genomic prediction of proteome fluctuation. (a) Box plot demonstrating the distribution of Spearman correlations for each gene with an associated eggNOG functional category. The Spearman correlation (ρ) between the summed metagenomic counts per million per time point and the average relative abundance of associated metaproteomic protein is displayed. Summary statistics for these data can be found in Table S1. (b) Summed GO categories with strong genomic and proteomic correlation. (c) Summed GO categories with weak genomic and proteomic correlation.

Because proteins have consistent roles (41), we expected that certain functional categories would show a stronger correlation between gene content and protein expression. We tested this hypothesis using several different functional databases for a comprehensive analysis. After removing human proteins and subdividing individual genes by functional category (evolutionary genealogy of genes: nonsupervised orthologous groups [eggNOG]), the distribution of the data representing gene-to-protein Spearman correlations was largely consistent with the overall mean ρ value of ∼0.3 (Fig. 3a). Categories with the largest number of features shared, such as “Energy production and conversion,” “Carbohydrate transport and metabolism,” and “Translation, ribosomal structure, and biogenesis,” all had distributions centered near the Spearman ρ value of ∼0.3. Other categories with fewer features had more variability in their average correlation values. Less-abundant categories included “Cell cycle control,” which had a lower average correlation, and “Inorganic ion transport and metabolism,” which had a higher average correlation (Fig. 3a; see also Table S1 in the supplemental material). This indicates that there were no broad-scale functional group differences distinguishable from the overall low but positive correlation observed between all genes and proteins.

10.1128/mSystems.00337-18.6 TABLE S1 Summary statistics on the Spearman correlations between the genes shared in the metagenome and metaproteome by eggNOG category. Download Table S1, DOCX file, 0.03 MB. Copyright © 2019 Mills et al. 2019 Mills et al. This content is distributed under the terms of the Creative Commons Attribution 4.0 International license.

In addition to individual gene correlations, we also evaluated inter-omic relationships between the abundances of entire gene categories. We assessed these relationships through summing protein and gene abundances by GO annotation and performing Spearman correlations (Table S2). There was large variability (σ = 0.445) in the correlations of different functional groupings with an average Spearman ρ value of 0.135. Despite the low overall correlation, themes of GO categories with similar correlations were present. Several GO terms related to polysaccharide, formate, and anaerobic respiration all had strong positive correlations above ρ = 0.6 (Fig. 3b). Other categories had consistently low or even negative correlations below ρ = 0.2. Cell wall and membrane proteins and metal binding proteins and chaperones were among the categories with poor correlations (Fig. 3c). These results suggest that there are some categories of genes that better represent protein expression levels, which may be the result of constitutive versus inducible expression. However, the techniques used also influence particular categories, such as membrane proteins, whose hydrophobic nature presents a challenge to MS workflows (42). All of the described categories had greater than 200 proteins and genes contributing to these relationships, which indicates that the findings was not related to differences based on the presence of high- or low-abundance proteins.

10.1128/mSystems.00337-18.7 TABLE S2 Summed GO inter-omic correlations and dynamic ranges. Download Table S2, XLSX file, 2.1 MB. Copyright © 2019 Mills et al. 2019 Mills et al. This content is distributed under the terms of the Creative Commons Attribution 4.0 International license.

### Taxonomic correlations with inflammatory markers are largely shared at the protein and gene levels.

**FIG 4:**
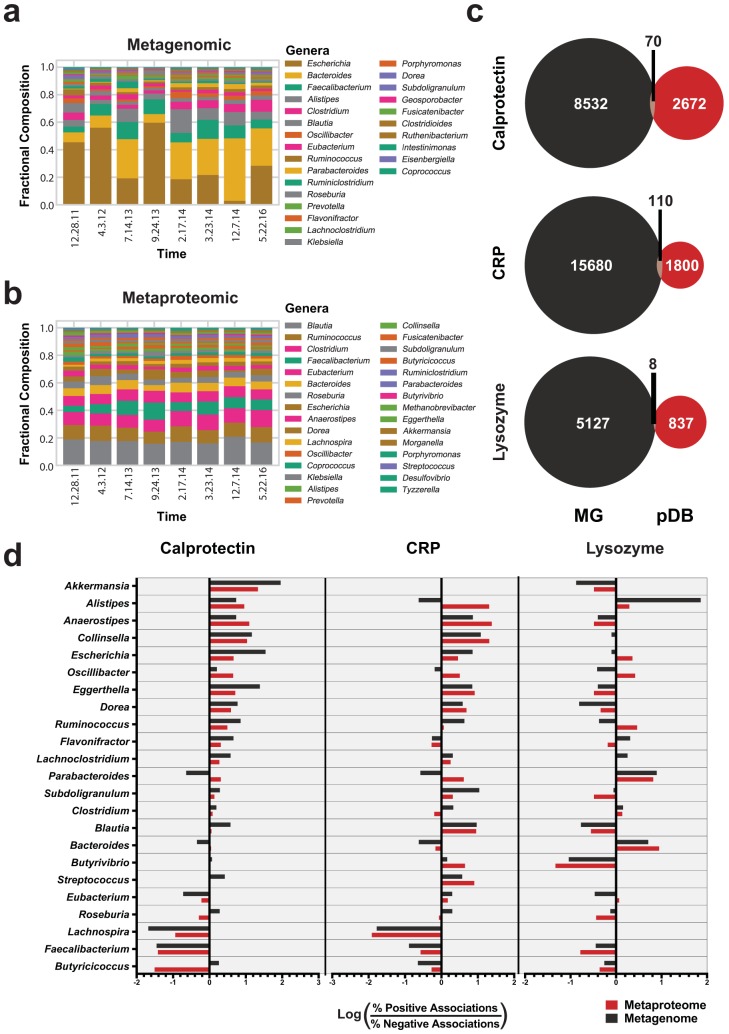
Genus-level associations with clinical markers. (a and b) Bar plot displaying the fractional composition of the most abundant genera (>0.03) in the metagenome (a) and the metaproteome (b) in each of the samples analyzed. (c) Comparison of genes and proteins significantly associated with each clinical marker. Venn diagrams show the number of genes and proteins with a large effect size (|*r*| > 0.7) with respect to clinical markers based on linear regression. (d) Genera associated with clinical markers. The associated proteins with genus-level taxonomy analyzed as described for panel c were compared by determining the log ratios of the compositions of proteins with positive and negative associations. The log ratio is plotted on the *x* axis for each clinical marker, and bars represent the association with each genus. Metaproteome values are plotted in red, and metagenome values are plotted in black. The numbers of genes and proteins included in this analysis are listed in Table S2.

We next sought to determine whether fluctuations related to inflammatory markers were conserved between genes and proteins. Taxonomic assignments for the pDB database were assigned based on the protein sequences to ensure consistent assignments for both data sets. Genus-level compositions were significantly different in the metagenome but not in the metaproteome (Friedman test *P* = 8.9e−5 and 0.69, respectively) (Fig. 4a and b). Dominant genera included *Escherichia*, *Bacteroides*, *Faecalibacterium*, and *Alistipes* (Fig. 4a). For easier interpretation of the abundances used for metagenome comparisons, the metaproteome composition was intentionally not adjusted for the lowest common ancestor of the peptides (43). Metaproteome taxonomic composition plots adjusted for lowest common ancestor also displayed stable compositions, though certain genera, such as *Blautia*, had a notably different composition after the adjustment (Fig. S2a-b).

10.1128/mSystems.00337-18.2 FIG S2 Taxonomic relationships to disease severity. (a) Lowest common ancestor-adjusted phylum-level composition of metaproteome data. (b) Lowest common ancestor-adjusted genus-level composition of metaproteome data. (c) Correlations between the microbial dysbiosis disease index and clinical markers of severity. Best-fit lines are displayed surrounded by 95% confidence intervals. Download FIG S2, PDF file, 1.2 MB. Copyright © 2019 Mills et al. 2019 Mills et al. This content is distributed under the terms of the Creative Commons Attribution 4.0 International license.

To evaluate the relationship between species related to inflammation in CD and our biomarkers of interest, we evaluated each immune protein against a previously defined microbial dysbiosis index (19). This index was developed using hundreds of samples from both Crohn’s disease patients and healthy controls to predict CD severity through analysis of log ratios of the species that were increased and decreased in abundance within CD (19). Nineteen of the species defined in the index were found in our data set. These included Escherichia coli and Fusobacterium nucleatum, which are increased in abundance in CD, and Faecalibacterium prausnitzii, Eubacterium rectale, and Bacteroides vulgatus, which are decreased in abundance in CD. After summing gene and protein abundances and determining the relationship between log ratios and each biomarker, fecal calprotectin was found to have the strongest association with the microbial dysbiosis index in both the metagenome and metaproteome. This result was not statistically significant, which was likely a result either of the small sample size or of the extrapolation of methods developed from hundreds of patients for use with a single subject (Fig. S2c).

Linear regression analyses were performed against inflammatory markers on each gene and protein. To evaluate our results, we compared the positively and negatively associated genes with large effect sizes (44) (correlation coefficient, |*r*| > 0.7). Interestingly, most of the individual genes and proteins associated with each of the inflammatory markers were unique, with only 0.5% (188/34,836) of associations shared between data types (Fig. 4c). Accounting for only the genes and proteins quantified in both data sets, 10% (188/1,814) of the strong associations were shared between data sets (Fig. S3).

10.1128/mSystems.00337-18.3 FIG S3 Comparison of highly correlated proteins and genes from shared identifications. The Venn diagrams represent the overlapping genes and proteins from the 3,598 ORFs representing both gene counts and protein abundances. Genes and proteins were deemed highly correlated by effect size (|*r*| > 0.7) after calculations of linear regressions with respect to the abundance of each clinical marker were performed (a, calprotectin; b, CRP; c, lysozyme). Red, metaproteome; black, metagenome. Download FIG S3, PDF file, 0.3 MB. Copyright © 2019 Mills et al. 2019 Mills et al. This content is distributed under the terms of the Creative Commons Attribution 4.0 International license.

Despite the lack of overlap in the individual identities of the genes and proteins correlated with each clinical marker, we observed consistent trends in the taxonomic annotations among the correlated genes and proteins. With over 800 genes and proteins strongly correlated to each marker (|*r*| > 0.7), we contrasted the taxonomic compositions of the positive and negative correlations. Several genera had >30-fold differences between compositions (Fig. 4d). Genus-level differences were largely conserved between data types in both direction and magnitude of association (Fig. 4d). *Akkermansia* and *Anaerostipes* had the strongest proinflammatory relationship whereas *Faecalibacterium* and *Butyricicoccus* had the largest anti-inflammatory relationship as assessed through the number of proteins positively or negatively correlated to calprotectin (Fig. 4d). Several genus-level trends such as those corresponding to *Alistipes*, *Anaerostipes*, *Faecalibacterium*, and *Lachnospira* were conserved between CRP and calprotectin, while lysozyme had largely different associated genera. Contextually, the number of proteins and genes used to generate these associations is important for the interpretation of these results as some associations were based on very few observations (Table S3).

10.1128/mSystems.00337-18.8 TABLE S3 Total number of proteins and genes significantly correlated to each of the genera. Download Table S3, DOCX file, 0.03 MB. Copyright © 2019 Mills et al. 2019 Mills et al. This content is distributed under the terms of the Creative Commons Attribution 4.0 International license.

Lysozyme is a component of the innate immune response that targets Gram-positive cell walls. Interestingly, proteins and genes correlated with lysozyme levels had large phylum-level changes (Fig. S4a). *Bacteroidetes* is a Gram-negative phylum, while *Firmicutes* is largely a Gram-positive phylum (45). The Gram-positive *Firmicutes* were enriched 1.4-fold among negative associations with lysozyme in both gene and proteins, while the Gram-negative *Bacteroidetes* were enriched 4.3-fold and 8.9-fold among positively correlated proteins and genes, respectively (Fig. S4a). Even though there were more than 800 genes and proteins from *Firmicutes* and *Bacteroidetes* that were correlated to lysozyme, very few from other phyla, such as the Gram-negative *Proteobacteria* and Gram-positive *Actinobacteria*, were observed. To validate these observations at the genus level, Gram staining information was cross-referenced (46). Although there were genera with both Gram-negative and Gram-positive species, the genus-level associations with lysozyme largely reflected the phylum-level observations (Fig. S4b).

10.1128/mSystems.00337-18.4 FIG S4 Taxonomic relationships to lysozyme. (a) The compositions of highly correlated proteins and genes related to lysozyme levels (|*r*| > 0.7) were compared, and the log ratio per phylum was plotted at the metagenomic and metaproteomic levels. (b) Proteins and genes used as described for panel a were analyzed at the genus level. Genera were grouped by Gram staining results. Download FIG S4, PDF file, 0.4 MB. Copyright © 2019 Mills et al. 2019 Mills et al. This content is distributed under the terms of the Creative Commons Attribution 4.0 International license.

### Comparing functional interpretations of the genes and proteins associated with immunological biomarkers.

**FIG 5:**
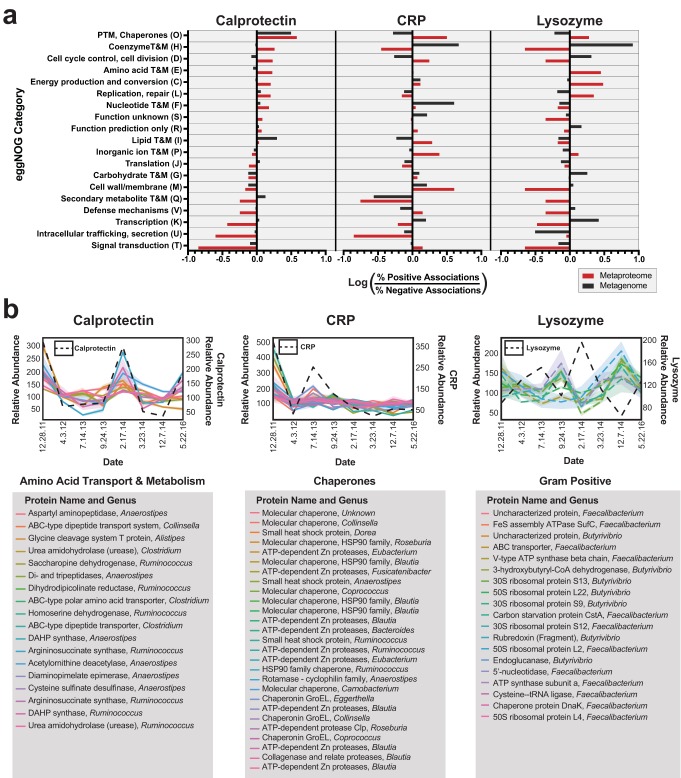
Functional associations with clinical markers. (a) Functions associated with clinical markers. Linear regressions to clinical markers were performed and the number of proteins or genes derived from each functional group with a large effect size (|*r*| > 0.7) were compared. The log ratio of the composition of positive and negative proteins is plotted on the *x* axis for each clinical marker. Metaproteome values are plotted in red and metagenome values are plotted in black. PTM, posttranslational modification; T&M, transport and metabolism. (b) Time series plots of selected proteins of interest. Protein abundances of one finding from each clinical marker are shown. A legend describing the protein names and associated genera is shown below each graph. DAHP synthase, 3-deoxy-d-arabinoheptulosonate 7-phosphate synthase.

Using the same identifications from linear regressions that provided the genus-level results, we next compared broad-scale functional groupings. The broad-scale functional associations were weaker than the genus associations. This observation may represent the effects of broad-scale categorization versus fine-scale categorization. Illustrating this point, the largest difference among the associations of genera was 90-fold, while the largest difference between functional groupings using assignments to the eggNOG database was 12-fold (Fig. 4d; see also Fig. 5a). Analyzing a broader taxonomic category, we observed that the maximum difference among comparisons of phyla was 8.9-fold (Fig. S4a), considerably closer to the 12-fold maximum for eggNOG categories. An additional consideration with respect to this result is the annotation rate for functional assignments. Only 15% of observed ORFs had an identifiable function, and this lower annotation rate may bias the results.

Despite the weaker associations of functional categories, several functional relationships with the disease markers were of interest. In total, 19 eggNOG categories (12 from the metaproteome, 7 from the metagenome) had differences of 3-fold or greater (Fig. 5a). Comparing the categories with associations with different immune markers provided insight into how different data types might influence functional interpretation. For example, metagenomic data had several strong functional associations that were not confirmed by protein abundances. One such category, “Nucleotide transport and metabolism,” had 147 genes positively correlated with CRP and 0 genes negatively correlated, indicating a positive association with CRP. The metaproteome data for this category had almost no association with CRP (Fig. 5a), with 6 proteins negatively correlated and 38 proteins positively correlated. We suspect that nucleotide metabolism undergoes protein expression in a manner independent of inflammatory conditions. The underlying reasons for this observation need to be further investigated.

Biologically relevant relationships were observed in the metaproteome that were not detectable in the metagenome. Free amino acids and urease enzymes have previously been associated with gut dysbiosis and Crohn’s disease (47). Interestingly, the metaproteome data identified a functional association of amino acid metabolism proteins with calprotectin, while this observation was absent in the metagenomic data (Fig. 5a). This observation included several urease proteins, as well as transporters for free amino acids, many of which were derived from the genera that had positive associations with inflammation (Fig. 5b). These ureases and transporters thus represent interesting targets for further investigation and represent further evidence of a previously established connection (47).

Another observation that was exclusively related to the metaproteome data was the relationship of chaperone proteins to several of the inflammatory metrics. There were 15 chaperone proteins with similar trends in expression with respect to CRP (Fig. 5b). This corresponded to posttranslational modification and chaperone proteins having 3.2-fold-higher representation in positively associated proteins and 1.9-fold-lower representation in genes (Fig. 5a). This unique observation from our patient’s fecal metaproteome is a potential indication of microbial stress occurring in response to the acute phase response and may indicate a need for the microbiome to refold proteins.

Because lysozyme targets Gram-positive cell walls, we expected correlated genes and proteins to be influenced by taxonomy and to have functions related to cell walls or membranes. However, cell wall proteins were underrepresented in the metaproteomic data set relative to their occurrence in the metagenomic data set (Table S4). Of the cell wall proteins associated with lysozyme, two (COG1088 and COG0463) were related to cell wall biosynthesis, encoding a glycosyl transferase and a dTDP-glucose 4-6-dehydratase. In this case, the binding of lysozyme to peptidoglycan may have disrupted the binding of these cell wall/membrane/envelope biogenesis proteins, leading to the observed negative correlation. Even though we were not able to detect many membrane or cell wall proteins related to lysozyme, 19 negatively correlated proteins from the butyrate-producing (48), Gram-positive genera *Faecalibacterium* and *Butyrivibrio* were identified (Fig. 5b). These proteins included 6 ribosomal proteins, which may indicate decreased translation occurring in the presence of lysozyme.

10.1128/mSystems.00337-18.5 FIG S5 Analysis of other immune biomarkers. (a) Other immune markers (lactotransferrin and secretory IgA) showed trends similar to those seen with calprotectin. The proteome relative abundances of IGHA1 and lactotransferrin and calprotectin are displayed for each time point. (b) The genus-level associations with lactotransferrin were similar to those seen with calprotectin. Calculations of linear regressions with respect to clinical markers were performed, and the numbers of highly correlated (|*r*| < 0.7) proteins or genes derived from each functional group were compared. The log ratios of the compositions of positive and negative proteins are plotted on the *x* axis for each clinical marker. Metaproteome values are plotted in red, and metagenome values are plotted in black. (c) The function-level associations determined for lactotransferrin were similar to those determined for calprotectin. The methods used for the determinations were described to those described for panel b, but eggNOG categories were compared. (d) Time series plots of selected proteins of interest. Proteins with GO terms that included iron are plotted with associated 68% confidence intervals. A legend describing the protein names and associated genera is shown to the right of the plot. Download FIG S5, PDF file, 1.7 MB. Copyright © 2019 Mills et al. 2019 Mills et al. This content is distributed under the terms of the Creative Commons Attribution 4.0 International license.

10.1128/mSystems.00337-18.9 TABLE S4 Total number of proteins and genes significantly correlated to each eggNOG category. Download Table S4, DOCX file, 0.03 MB. Copyright © 2019 Mills et al. 2019 Mills et al. This content is distributed under the terms of the Creative Commons Attribution 4.0 International license.

In addition to analyzing calprotectin, CRP, and lysozyme levels, we also evaluated S-IgA and lactoferrin levels. Secretory IgA is secreted in large quantities in the intestine to maintain favorable microbial compositions (49), and lactoferrin sequesters iron as an antimicrobial response (50). We observed similar expression patterns of lactoferrin, S-IgA, and calprotectin (Fig. S5a). The similar expression patterns resulted in minimal differences in both genus and functional relationships between calprotectin, lactoferrin, and S-IgA (Fig. S5b and c). Proteins positively associated with lactoferrin (|*r*| = >0.7) had a larger portion of GO terms related to iron (15.5% of 470 positive associations and 10% of 233 negative associations). Many of these proteins were pyruvate oxidoreductases, which are used in anaerobic bacteria for forming acetyl-coenzyme A (acetyl-CoA) from pyruvate (51) (Fig. S5d). These are crucial enzymes for certain anaerobic bacteria and have been suggested as potential drug targets (51). This result suggests that a connection exists between the iron-sequestering host proteins and the microbial proteins in our patient that are dependent on iron as a cofactor.

## DISCUSSION

Our investigation of the fundamental relationship between changes in the metagenome and the metaproteome revealed important considerations for interpreting these data types. Currently, studies using shotgun metagenomics to dissect the functions of the microbiome are becoming more prevalent (52), and the current study showed that differences at the gene level may not reflect differences at the protein level. Though discordance between RNA and protein expression is widely acknowledged for individual species (4), the relationships between DNA and protein content in the complex ecology of the microbiome are less understood. As these systems have rarely been studied in parallel, it is possible that communities of microbes influence the fundamental relationships between genes and proteins that had been previously established in monoculture settings. Although the metaproteomics field is improving in depth of coverage (8) and scope (13), the technical hurdles that MS presents often make DNA-based studies a more practical, higher-throughput solution. That being the case, functional insight from metagenomic studies requires a consideration of the relationship between protein abundances and metagenomic copy numbers.

Our results, although limited to a single patient, suggest that there is a degree of general agreement between changes in the metagenome and changes in the metaproteome. However, the relationship for individual genes/proteins is weak overall (our average Spearman ρ = 0.3). In the single-species context, bacterial systems have generally shown correlations between mRNA and proteins to range from ρ = 0.5 to 0.6 (38). Our experimental estimates indicate that DNA-to-protein correlations in complex microbial systems are notably lower. These associations do not appear to have obvious biases between large-scale functional groupings but do show certain trends in finer-resolution functional groupings such as individual GO terms. Representing an important notion in the field of IBD, formate- and nitrate-related categories had large fluctuations and consistent trends between the two data types. Formate oxidation has been implicated as a metabolic signature of inflammation-associated dysbiosis (53), indicating that metagenomic studies may predict protein abundances within this system. We do not believe that the consistency of the relationship between formate oxidation genes and proteins is a result of constitutive expression, as, at least in E. coli, related genes such as formate hydrogenase genes are regulated by the presence of formate (54). Nitrate-based anaerobic respiration is implicated in promoting the growth of facultative anaerobes such as the Enterobacteriaceae, which can lead to microbial dysbiosis and intestinal inflammation (45). Tables of the identified eggNOG and GO terms are provided and indicate how well the metagenomic copy number predicted the protein abundances within each identified category.

Identifying the genes and proteins with similar expression trends with respect to certain inflammatory and immune markers revealed that there were large differences in genus-level associations that were biologically relevant and generally consistent between data types. *Faecalibacterium* is a genus depleted in IBD (19, 55) and appears to have anti-inflammatory effects, possibly mediated by butyrate production (56). Both data types had a strong negative correlation in numerous *Faecalibacterium* proteins to our biomarker for local inflammation, calprotectin. While it was previously shown that there were consistent trends between these data types showing increased *Faecalibacterium* in healthy patients (23), our results show these relationships can occur within a patient through time in a manner that corresponds to the current level of inflammation. Other trends were also found for well-documented genera with inflammatory roles in IBD (19), including E. coli, which is of particular interest because of its adherent-invasive properties in CD (57, 58). Interestingly, these shared trends were found with almost entirely different genes. This may indicate that the underlying bacterial abundance influences both of these data types but that the individual proteins expressed at certain times are not directly associated with the amount of corresponding genetic material present. If this is the case, it is possible that functional associations made through some broad-scale categories, such as eggNOG, may have different results depending on the data type. This concept is supported by our results that indicate less-extensive and less-consistent associations with broad-scale groupings than with associations at the genus level.

Our analysis of clinical biomarkers was useful for understanding the biology associated with each immune component. As calprotectin had the strongest association with the microbial dysbiosis index (19), the results suggests that the level of calprotectin may be a better indication of microbial imbalances. Interestingly, CRP has been reported to represent a less useful diagnostic tool than fecal calprotectin for intestinal inflammation (29). CRP levels may be a better indication of systemic inflammation, and we observed here that the levels of many bacterial chaperone proteins may be increased in correspondence. We observed taxonomic trends with the abundances of lysozyme that were consistent with its biological function of acting upon cell walls. In general, predominately Gram-positive genera and phyla had a larger portion of anticorrelated genes and proteins, while Gram-negative bacteria had an opposite association.

Our observed discrepancies between gene and protein levels may have large implications for data interpretation, but it is important to replicate these results in a larger cohort of IBD patients. As certain GO categories present strong correlations between data types, it suggests that it may be possible to develop a metagenomic-metaproteomic reference guide for creating stronger functional hypotheses. This guide may be used to outline which groups of genes have a strong or weak association with protein abundances.

The relationship between genes and proteins may be influenced by several factors. Correlation between DNA and protein abundances might reflect the presence of DNA from dormant or dead cells (59), which may lead to a higher level of correlation (because the cells are not actively producing or secreting proteins). Other factors may include constitutive versus inducible genes or the stability of the proteins. For example, chaperone proteins were found in high abundance which may be a result of their high stability and of their stable concentrations within the cell (60). Ultimately, the associations between -omic data sets are influenced by the nature of the data collection techniques and normalization, and further benchmarking is necessary. Although, there are significant challenges in integrating multi-omic data types (61), further understanding these relationships is of paramount importance as the microbiome field progresses.

Our study presents several technical findings of interest. Leverage of the modern TMT-based LC-MS^3^ quantification platform provided a highly accurate quantification method for comparison with gene counts. Our workflow designed for mediating comparisons between metagenomic and metaproteomic data expands our knowledge of data type differences and acts as a bioinformatic and technological update to previous studies (23). Additionally, the use of technical triplicates validates the reproducibility of these methods and helped increase our confidence in the quantification values at both the metagenomic and metaproteomic levels. However, outside validation from other technological pipelines may be necessary to further understand these biological systems. Our results are also derived from a small number of samples from one patient, and the time points were spread over large time spans. This design provided unique opportunities but limits our interpretation of the data to a single individual.

From a biological perspective, our results provide evidence that certain proteins and genera are correlated or anticorrelated with immunoprotein markers of inflammation. While the taxonomic insights that we observed were conserved between data types, our functional interpretations differed. This personalized perspective also demonstrates the extent of variability occurring within an individual, an important consideration to control for in studies with larger cohorts. Taking the results together, our study investigated the relationships between metagenomic and metaproteomic methods and highlighted important considerations for interpretation of meta-omic data.

## MATERIALS AND METHODS

### Ethics statement.

The patient had stool samples collected by consent under two protocols: HRPP 141853 (American Gut Project) and HRPP 150275 (Evaluating the Human Microbiome). Both protocols were approved by the Human Research Protection Program (HRPP) of the University of California, San Diego. Written informed consent obtained from the patient concerning dissemination and scientific publication of the results is also included in the approved protocols.

### Longitudinal sample collection.

Naturally passed fecal samples were collected and immediately stored without buffer at −80°C. Eight samples were selected. A personal symptom log entry was generated at the time that each fecal sample was passed. Additionally, the weight and body mass index (BMI) of the patient were determined on the day associated with each sample.

### Generation of metagenomic reads.

Samples were extracted according to the Earth Microbiome Project (2) protocol using a Qiagen MagAttract PowerSoil DNA kit as previously described (62). Briefly, swabbed fecal material was plated into 96-well PowerBead DNA plates containing garnet beads. DNA extraction was performed once on each of the eight samples according to the manufacturer's instructions, with an additional incubation at 65°C for 10 min following the addition of lysis solution and immediately prior to shaking (Qiagen TissueLyser II; Qiagen catalogue 85300). Magnetic DNA purification was performed using a KingFisher Flex purification system. Then, whole-genome shotgun libraries were made using a Nextera DNA library preparation kit (Illumina, San Diego, CA, USA) and a 1:10 miniaturized-reaction volume. Unique barcodes were used per triplicate totaling 24 metagenomic samples. The median insert sizes by sample ranged from 183 bp to 366 bp. Libraries were sequenced using Illumina MiSeq paired-end (2 by 250 bp) sequencing, filling a total of one lane.

### Processing of metagenomic reads for a shared reference library (pDB).

Because typical metagenomics and metaproteomics workflows require a reference database, it was necessary to use a minimal approach to create from scratch a single reference database that could be used for both metagenomics and metaproteomics from the individualized data. All reads from the technical triplicates of each sample were concatenated. Next, the MEGAHIT alignment program (63) was utilized for assembling short reads into larger contigs. Assembled contigs were searched for possible coding regions through the program Prodigal (64). Next, the program Diamond (65) was used for gene alignment to the uniref50 database (66). Finally, the most likely uniref50 entry, determined through bitScore, was used for the functional annotations. KEGG orthology annotations were cross-referenced using GhostKOALA (67). Taxonomic assignments were determined by Diamond alignment (65) to an in-house library of microbial genomes. Taxonomy was assigned from the translated amino acid sequence of each predicted ORF in the pDB. This database was used as a reference database for both mass spectrometry data and sequencing data. Scripts used for data processing are available online (https://github.com/knightlab-analyses/Crohns-MG-MP-Comparisons).

### Generating copy numbers of metagenomic genes.

The program Salmon (68) was applied to determine the reads present for each gene from the pDB. First, an index was created with Salmon, inputting the pDB fasta file. Next, reads were aligned to this index in quasimapping mode for each of the 24 metagenomic samples. The results were represented in counts per million sequences, with missing values padded as zeroes.

### Protein abundances from the shared reference library (pDB).

The generation of mass spectra data is described below. Spectral data were searched against the pDB with a concatenated human reference library (https://www.uniprot.org/; accessed 28 November 2016) using Proteome Discoverer 2.1 (Thermo Fisher Scientific). Further data processing is described below.

### Protein digestion and TMT labeling.

Fecal samples were measured out to ∼0.5 g and suspended in 10 ml of ice-cold, sterilized Tris-buffered saline (TBS). Samples were suspended through vortex mixing and homogenized through the use of a blender apparatus. A Steriflip (Millipore) filter (20 μM vacuum) was used to remove particulate from the samples. Cells were pelleted through centrifugation at 4,000 rpm for 10 min. Next, cells were lysed in 2 ml of buffer containing 75 mM NaCl (Sigma), 3% sodium dodecyl sulfate (SDS; Fisher), 1 mM NaF (Sigma), 1 mM beta-glycerophosphate (Sigma), 1 mM sodium orthovanadate (Sigma), 10 mM sodium pyrophosphate (Sigma), 1 mM phenylmethylsulfonyl fluoride (PMSF; Sigma), 1× Complete Mini EDTA free protease inhibitors (Roche), and 50 mM HEPES (Sigma), pH 8.5 (69). An equal volume of 8 M urea–50 mM HEPES (pH 8.5) was added to each sample. Cell lysis was achieved through two 10-s intervals of probe sonication at 25% amplitude. Proteins were then reduced with dithiothreitol (DTT; Sigma), alkylated with iodoacetamide (Sigma), and quenched as previously described (70). Proteins were then precipitated via chloroform-methanol precipitation, and the protein pellets were dried (71). Protein pellets were resuspended in 1 M urea–50 mM HEPES (pH 8.5) and digested overnight at room temperature with LysC (Wako) (72). A second, 6-h digestion was performed using trypsin at 37°C, and the reaction was stopped through addition of 10% trifluoroacetic acid (TFA; Pierce). Samples were then desalted through the use of C_18_ Sep-Paks (Waters) and eluted with 40% and 80% acetonitrile solutions containing 0.5% acetic acid (73). Concentrations of desalted peptides were determined with a bicinchoninic acid (BCA) assay (Thermo Scientific). Aliquots (50 μg) of each sample were dried in a SpeedVac, additional bridge channels consisting of 25 μg from each sample were created, and 50-μg aliquots of this solution were used in duplicate per TMT 10-plex as previously described (16). These bridge channels were used to control for labeling efficiency, interrun variation, mixing errors, and the heterogeneity present in each sample (74). Each sample or bridge channel was resuspended in 30% dry acetonitrile–200 mM HEPES (pH 8.5) for TMT labeling with 7 μl of the appropriate TMT reagent (14). Reagents 126 and 131 (Thermo Scientific) were used to bridge between MS runs. The remaining reagents were used to label samples in random order. Labeling was carried out for 1 h at room temperature and quenched by adding 8 μl of 5% hydroxylamine (Sigma). Labeled samples were acidified by adding 50 μl of 1% TFA. After TMT labeling, the products of the 10-plex experiments were combined, desalted through the use of C_18_ Sep-Paks, and dried using a SpeedVac.

### Basic pH reverse-phase liquid chromatography sample fractionation.

Sample fractionation was performed by basic pH reverse-phase liquid chromatography with concatenated fractions as previously described (75). Briefly, samples were resuspended in 5% formic acid–5% acetonitrile and separated over a C_18_ column (Thermo Scientific) (4.6 mm by 250 mm) on an Ultimate 3000 high-performance liquid chromatography (HPLC) system fitted with a fraction collector, degasser, and variable-wavelength detector. The separation was performed over a 22% to 35%, 60-min linear gradient of acetonitrile–10 mM ammonium bicarbonate (Fisher) at 0.5 ml/min. The resulting 96 fractions were combined as previously described (75). Fractions were dried under vacuum and resuspended in 5% formic acid–5% acetonitrile and analyzed by liquid chromatography (LC)-MS^2^/MS^3^ for identification and quantitation.

### LC-MS^2^/MS^3^ for protein identification and quantitation.

All LC-MS^2^/MS^3^ experiments were carried out on an Orbitrap Fusion mass spectrometer (Thermo Fisher Scientific) with an in-line EASY-nLC 1000 instrument (Thermo Fisher Scientific) and a chilled autosampler. Separation and acquisition settings were as previously defined (76).

### Proteomic data processing.

Data were processed using Proteome Discoverer 2.1 (Thermo Fisher Scientific). MS^2^ data were searched against the pDB and Uniprot human database (https://www.uniprot.org/; accessed 28 November 2016). The Sequest searching algorithm (77) was used to align spectra to database peptides. A precursor mass tolerance of 50 ppm (78, 79) and 0.6-Da tolerance were specified for the MS^2^ fragments. Static modification of TMT 10-plex tags on lysine and peptide N termini (+229.162932 Da), carbamidomethylation of cysteines (+57.02146 Da), and variable oxidation of methionine (+15.99492 Da) were included in the search parameters. Raw data were searched at a peptide and protein false-discovery rate (FDR) of 1% using a reverse-database-search strategy (80–82).

TMT reporter ion intensities were extracted from MS^3^ spectra for quantitative analysis, and signal-to-noise values were used for quantitation. Additional stringent filtering was used, removing any moderate-confidence peptide spectral matches (PSMs) or ambiguous PSM assignments. Additionally, any peptides with a spectral interference level above 25% were removed, as well as any peptides with an average signal-to-noise ratio of less than 10. In accordance with false discovery rate benchmarking (83), proteins matching only one high-confidence PSM were not removed. As metaproteome data contain a high degree of similarity in levels of identity between proteins, several decisions were made to reduce false assignments. Standardized methods in Proteome Discoverer (Version 2.1) preferentially assign peptides to proteins that had previously had peptides reported. If this does not resolve the assignment, the peptide is assigned to the longest protein. Additionally, a duplicate peptide filter was applied according to the Proteome Discoverer report. Normalization occurred as previously described (76). Briefly, relative abundances are normalized first to the pooled standards for each protein and then to the median signal across the pooled standard. An average of these normalizations was used for the next step. To account for slight differences in the amounts of protein labeled, these values were then normalized to the median of the entire data set and reported as final normalized summed signal-to-noise ratios per protein per sample.

### Use of an integrated gene catalog for reference library comparison.

The integrated reference catalog was downloaded from http://meta.genomics.cn/meta/home (accessed 22 December 2016). A two-step database search method was utilized (12). Briefly, the full database was used as a first-pass screen. Second, both forward and reverse database identifications were used to create a study-specific database. This database was used to search mass spectrometry data, and identifications were filtered at a 1% FDR for peptides and proteins.

### Data analysis.

Data analysis was performed in python version 3.5 (https://www.python.org/), and records of the code are available in corresponding Jupyter Notebooks for this project (https://github.com/knightlab-analyses/Crohns-MG-MP-Comparisons). All displayed metaproteomic data were generated using the pDB metaproteomic data unless otherwise specified. Qiime was used for principal-coordinate analysis (37). Spearman correlations were performed through the use of the pandas python package (http://pandas.pydata.org/). Linear regressions were performed on metagenome sums and metaproteome averages against the metaproteome abundances of each of the biomarker abundances. Protein and gene associations were ranked by the associated coefficient of correlation, and taxonomic and functional annotations of the top associated genes and proteins (|*r*| < 0.7) were compared. Linear regressions were performed using the python package scipy (https://www.scipy.org). Friedman tests were also performed through scipy, comparing genus compositions within the metagenome and metaproteome between samples.

### Data availability.

Proteomic data and supplementary files are available online at https://massive.ucsd.edu/ProteoSAFe/static/massive.jsp (study identifier [ID] MSV000082113). Metagenomic data are available through the European Bioinformatics Institute (EBI) (https://www.ebi.ac.uk/ena) under the study identifier PRJEB28712 (ERP110957).
