# Supplementary figures and images for: Evaluating Metagenomic Prediction of the Metaproteome in a 4.5-Year Study of a Patient with Crohn's Disease
Source: mSystems. 2019 Feb 12;4(1):e00337-18. doi: 10.1128/mSystems.00337-18 (PMC6372841; doi:10.1128/mSystems.00337-18)

**a**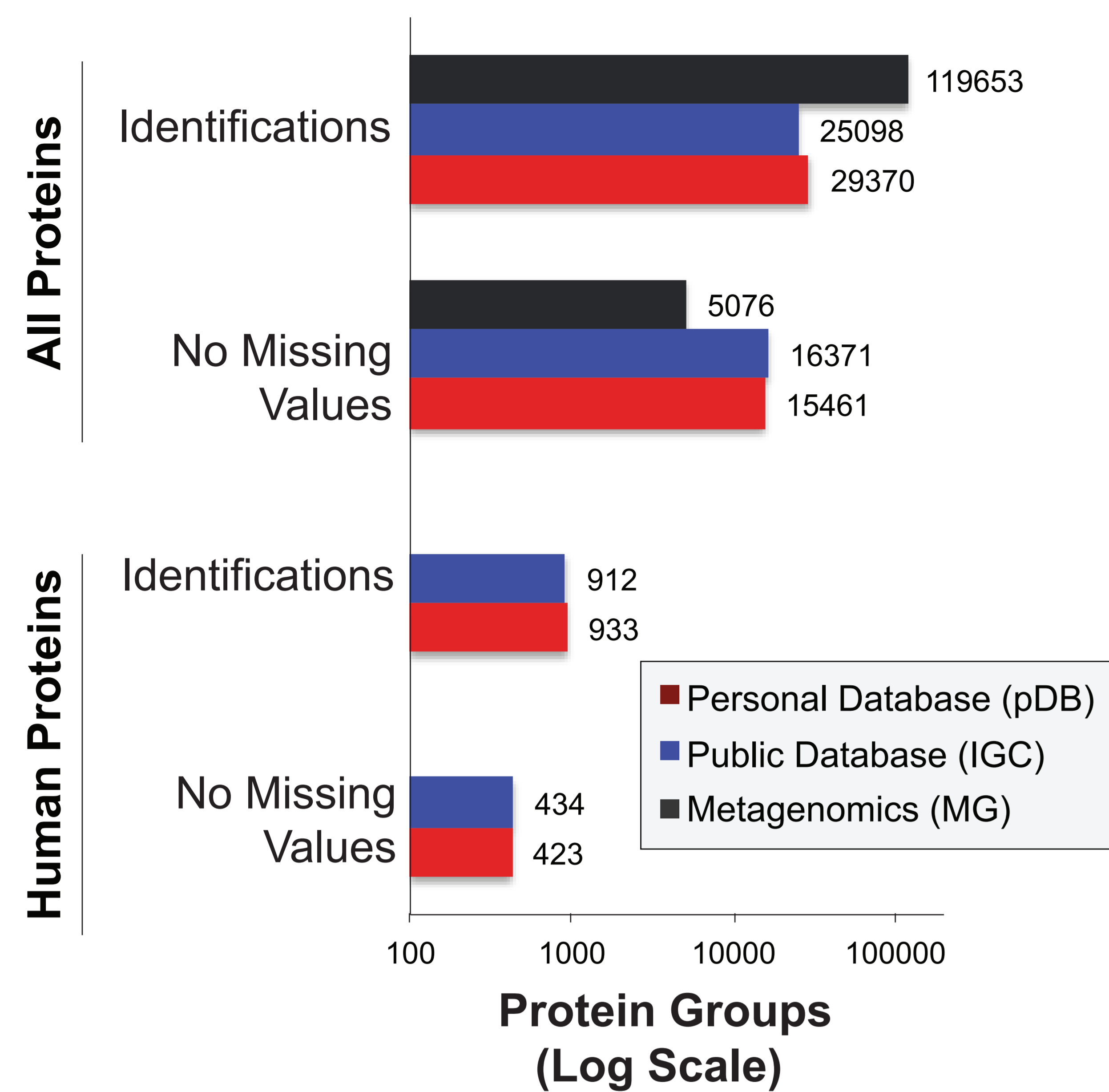**b**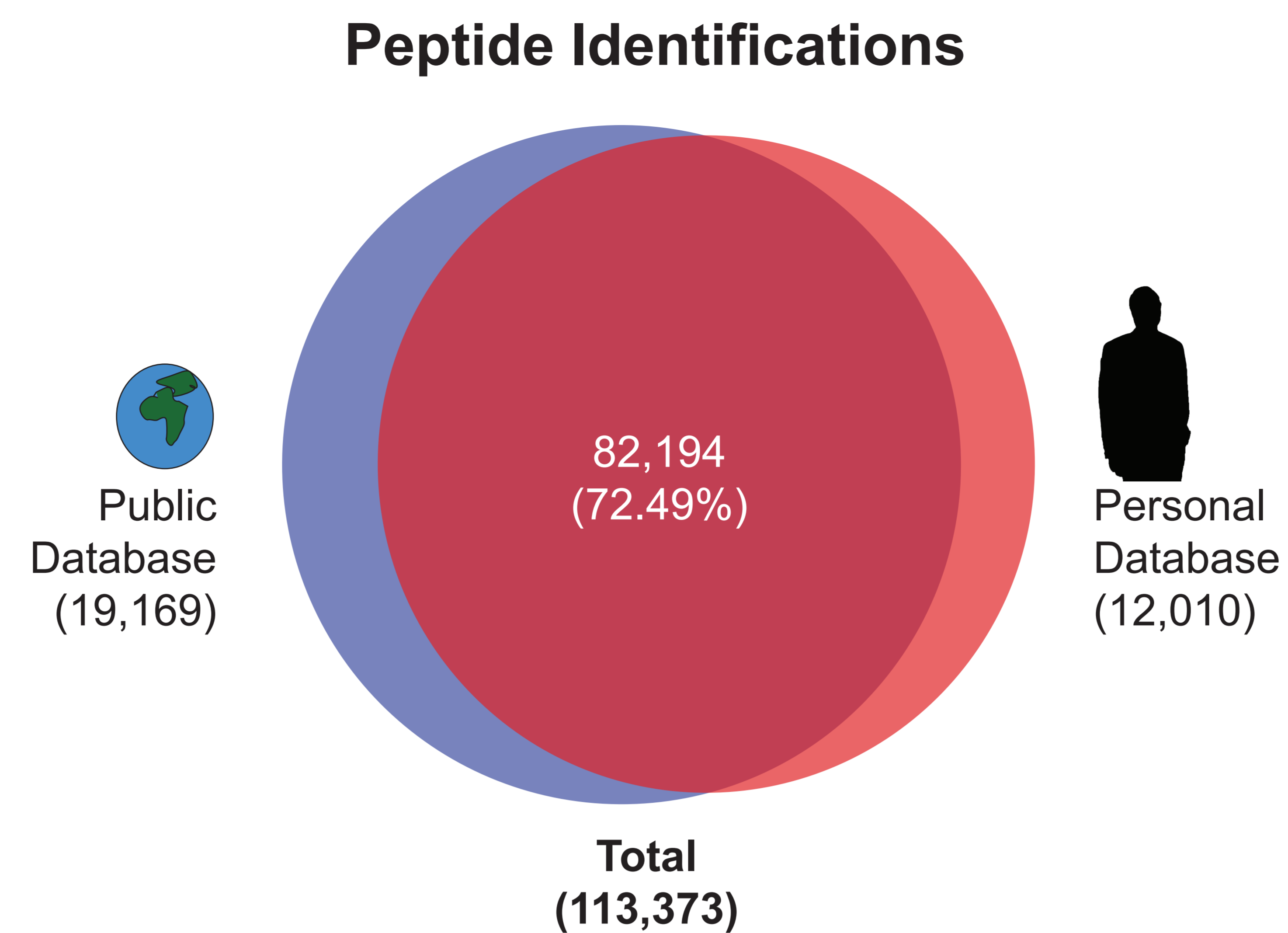**c**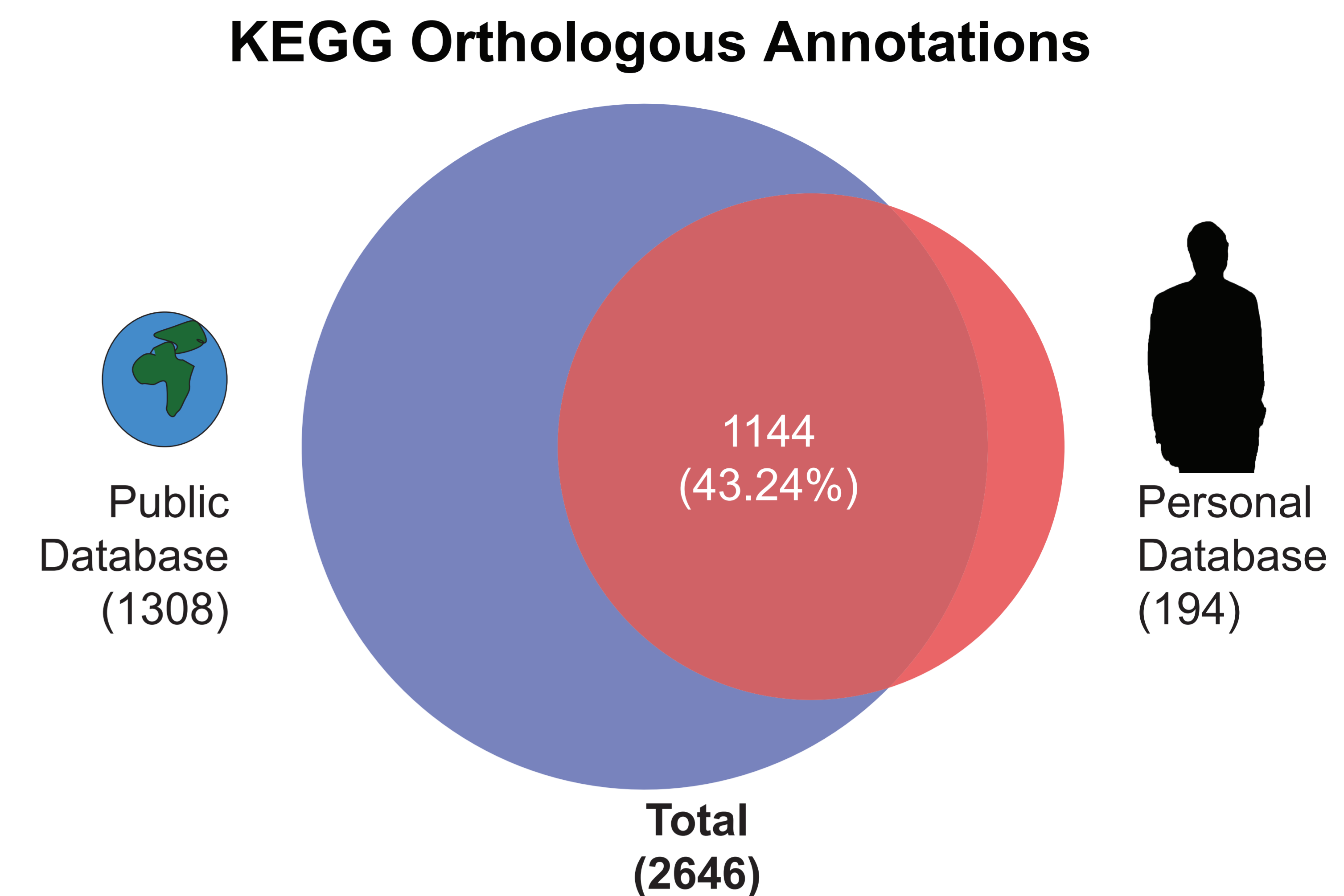**d**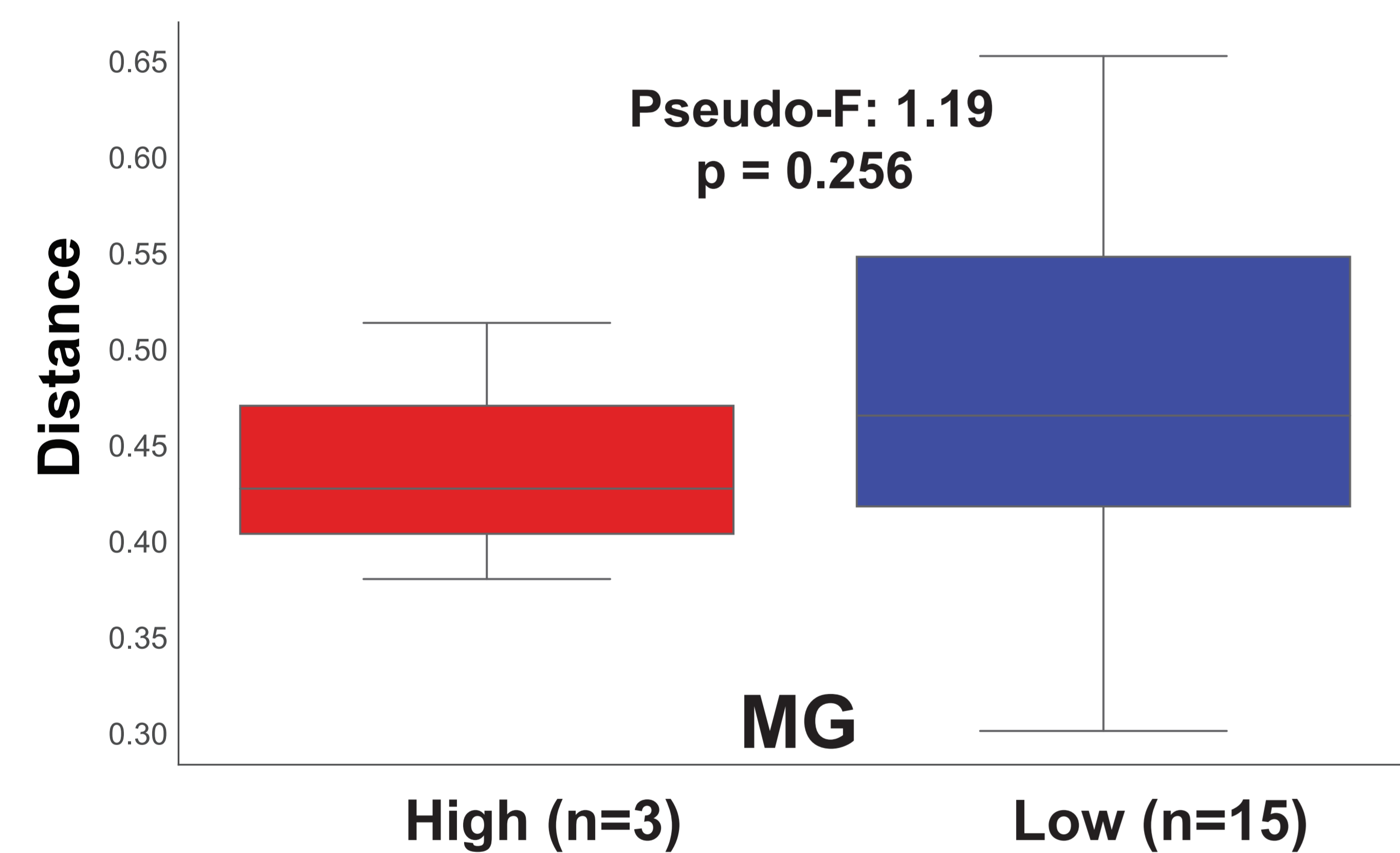**e**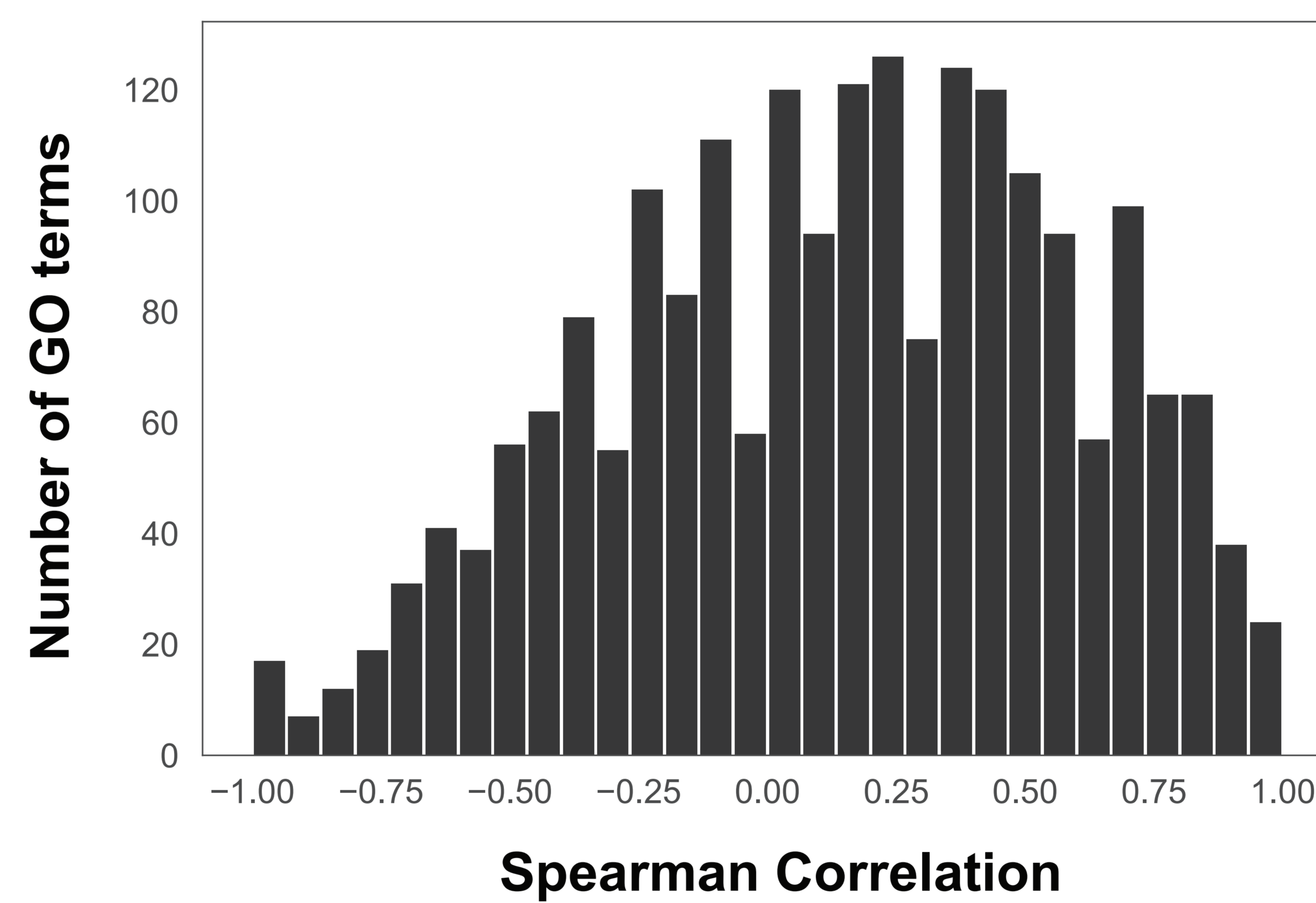**f**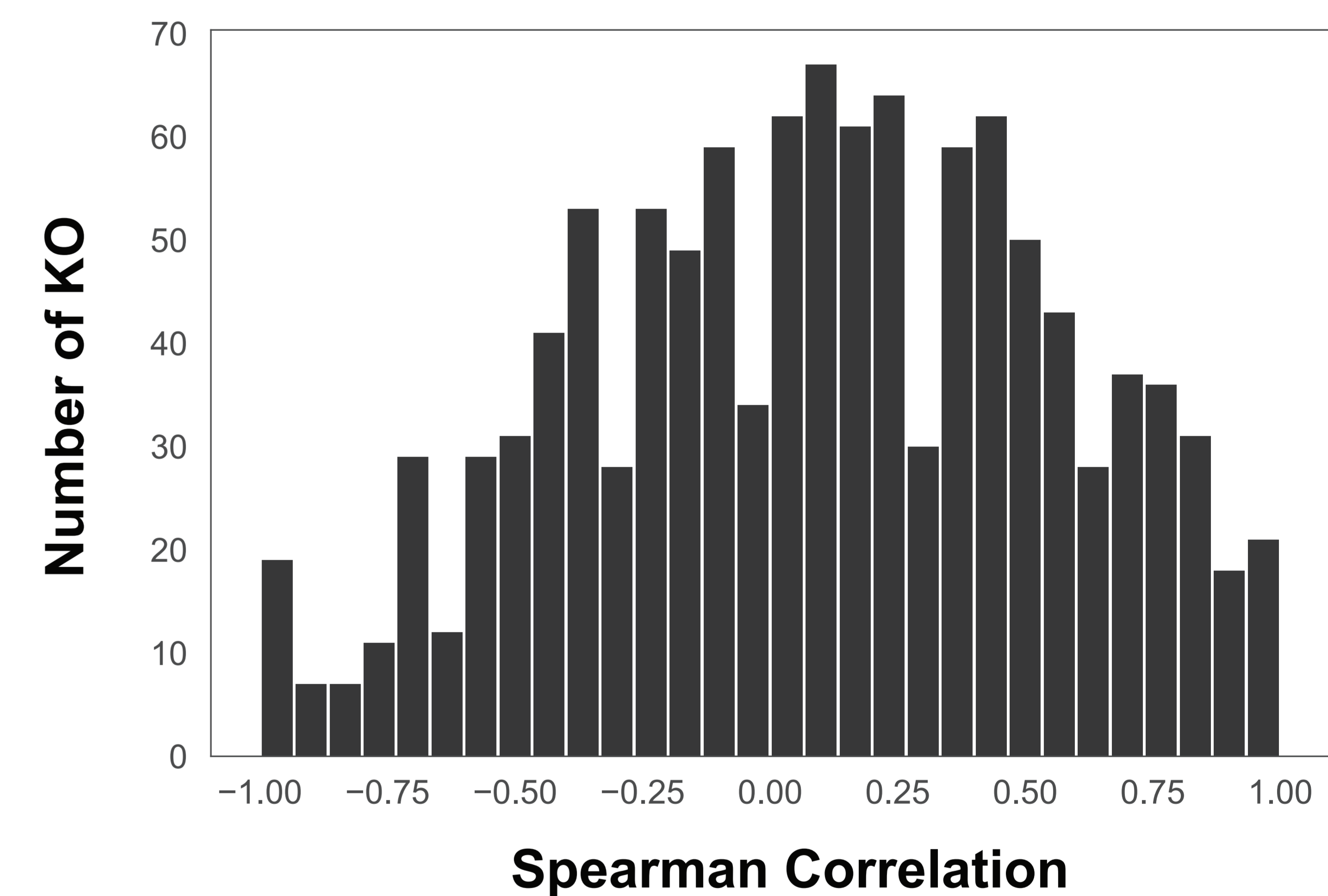**g**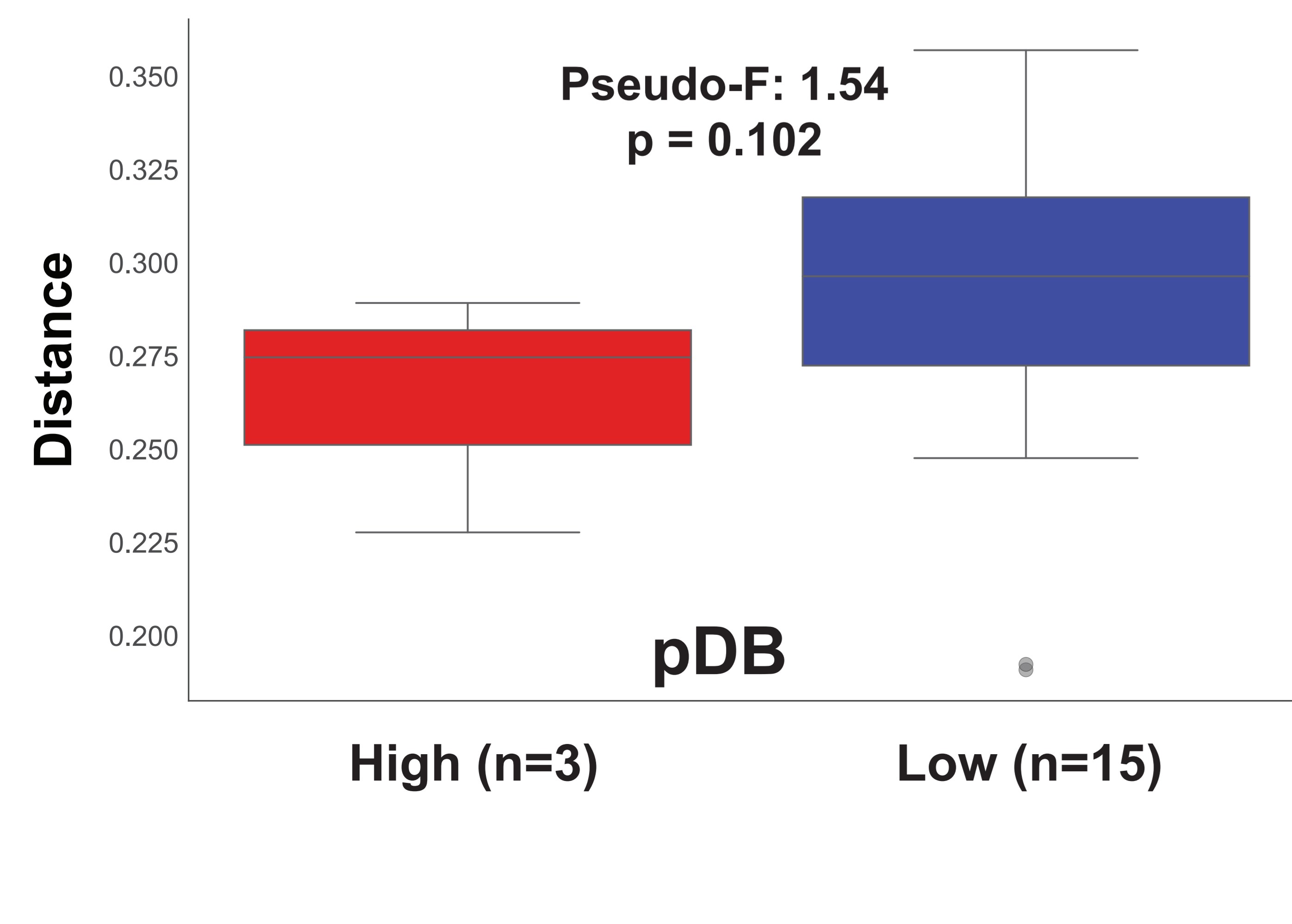**h**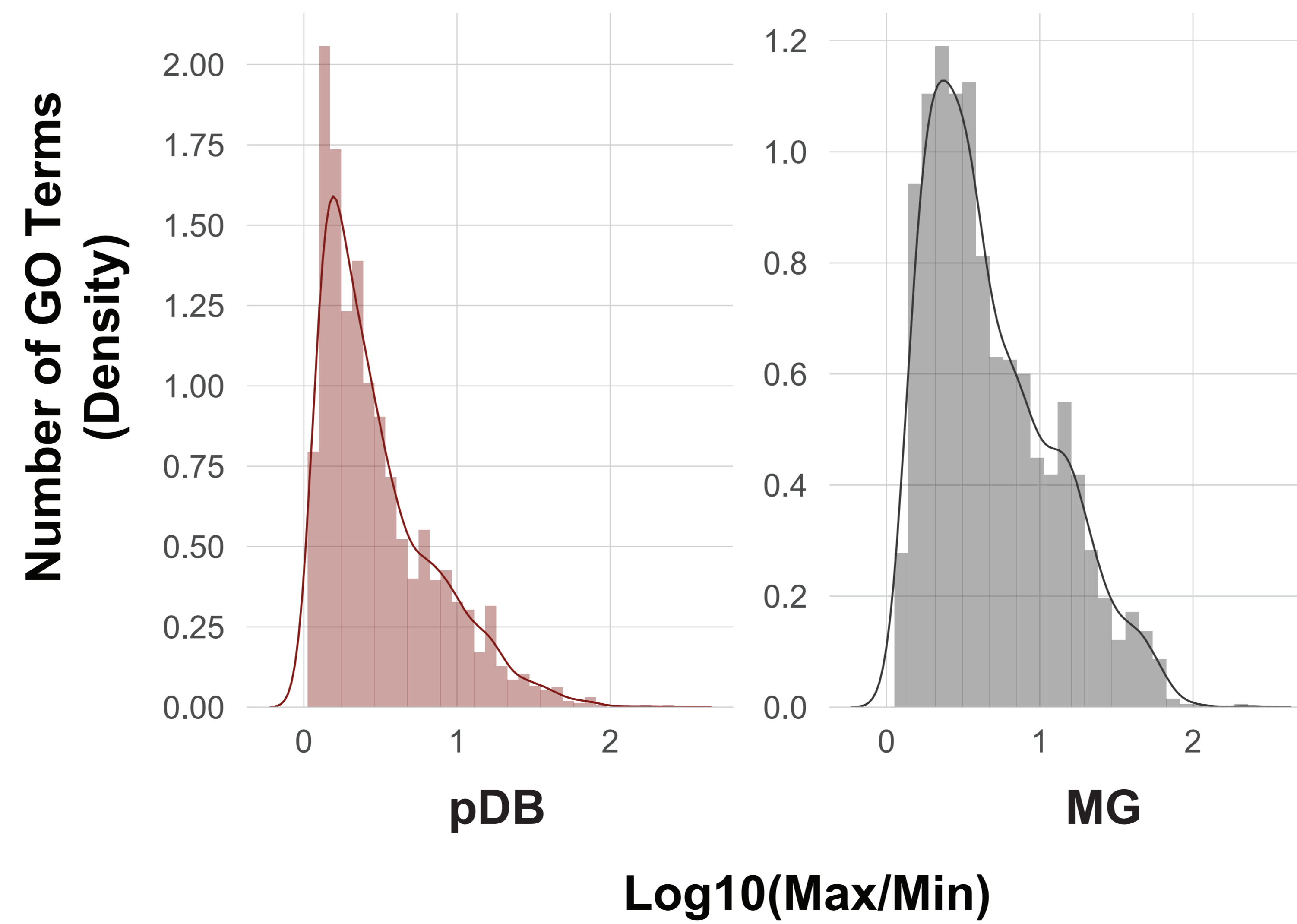**i**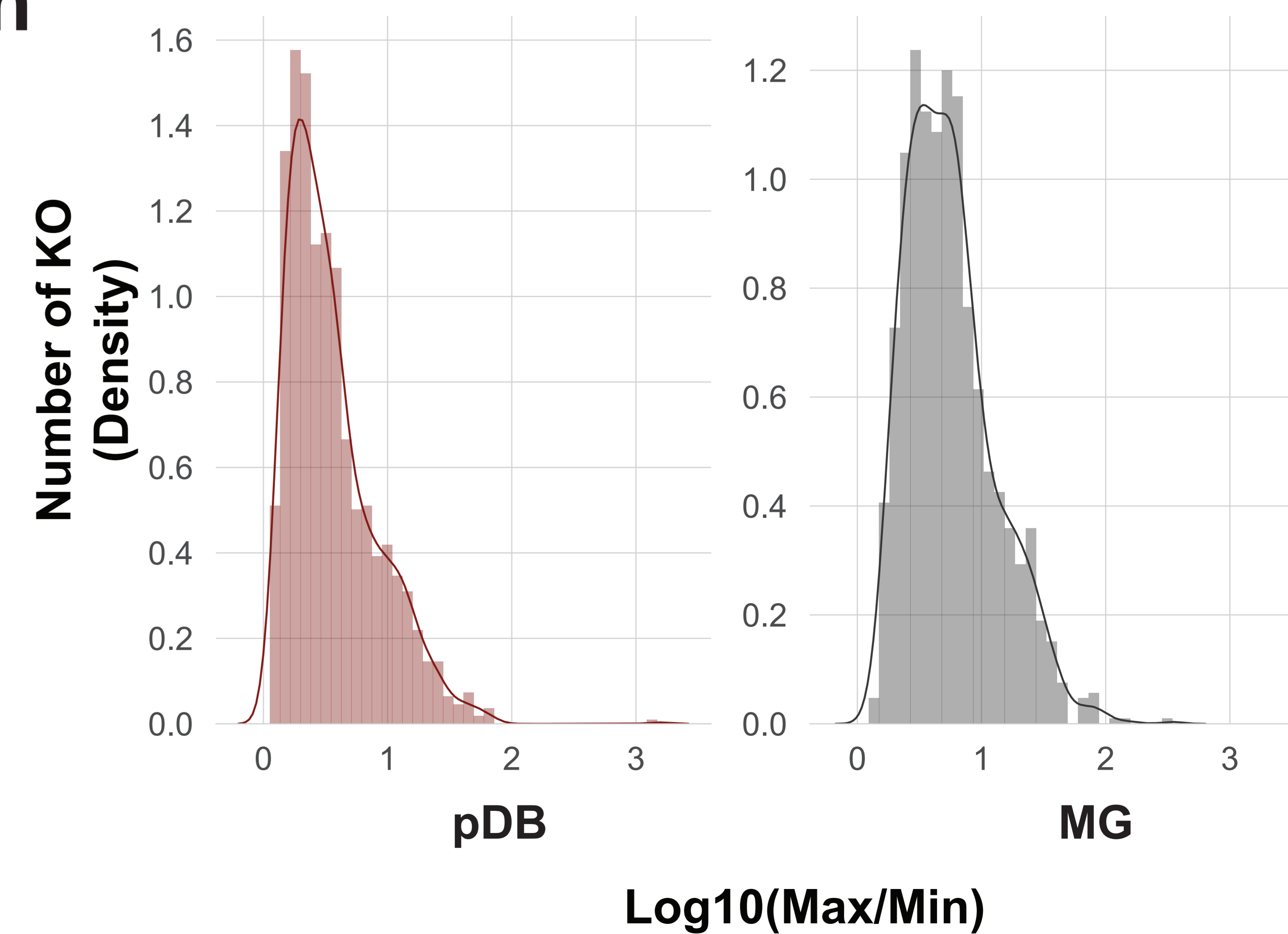

Supplement: FIG S1 [file mSystems.00337-18-sf001.pdf]

**a****Calprotectin**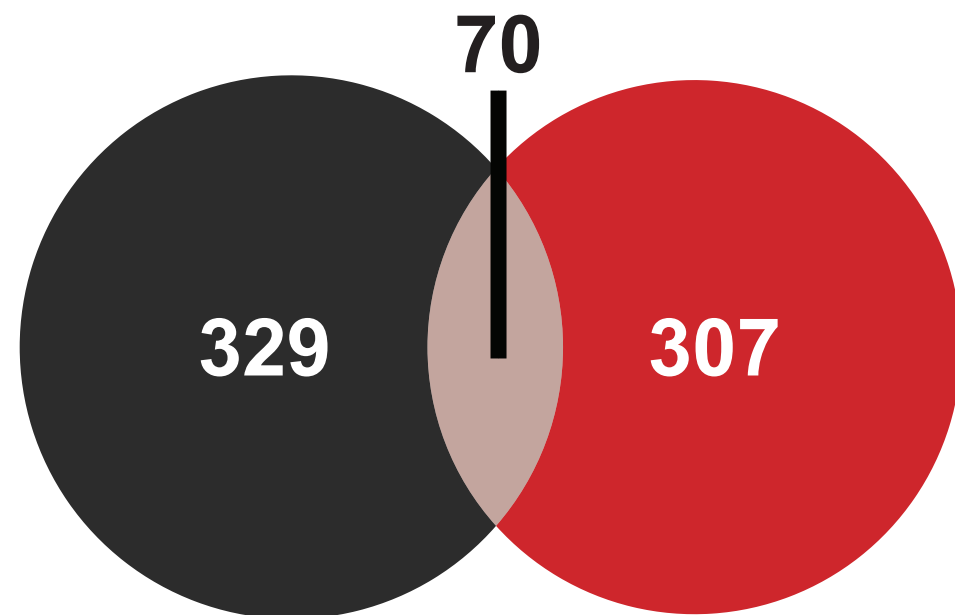**b****CRP**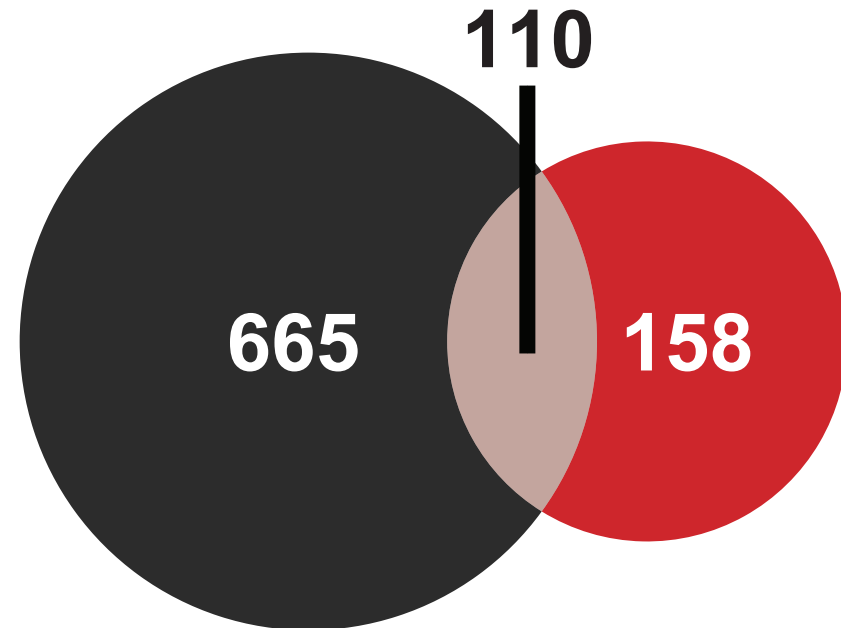**c****Lysozyme**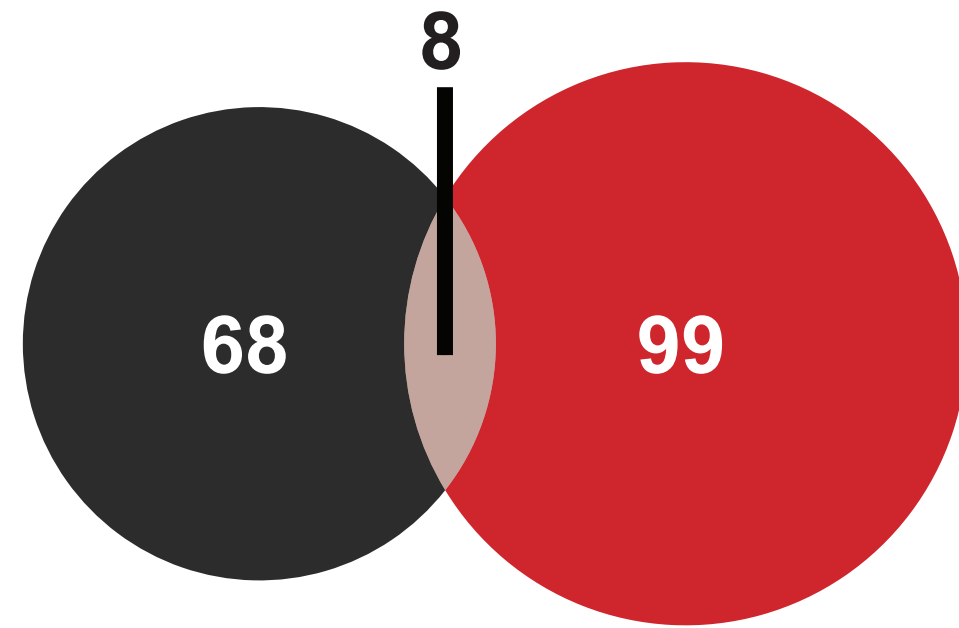

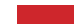 **Metaproteome**  
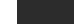 **Metagenome**

Supplement: FIG S3 [file mSystems.00337-18-sf003.pdf]

**a**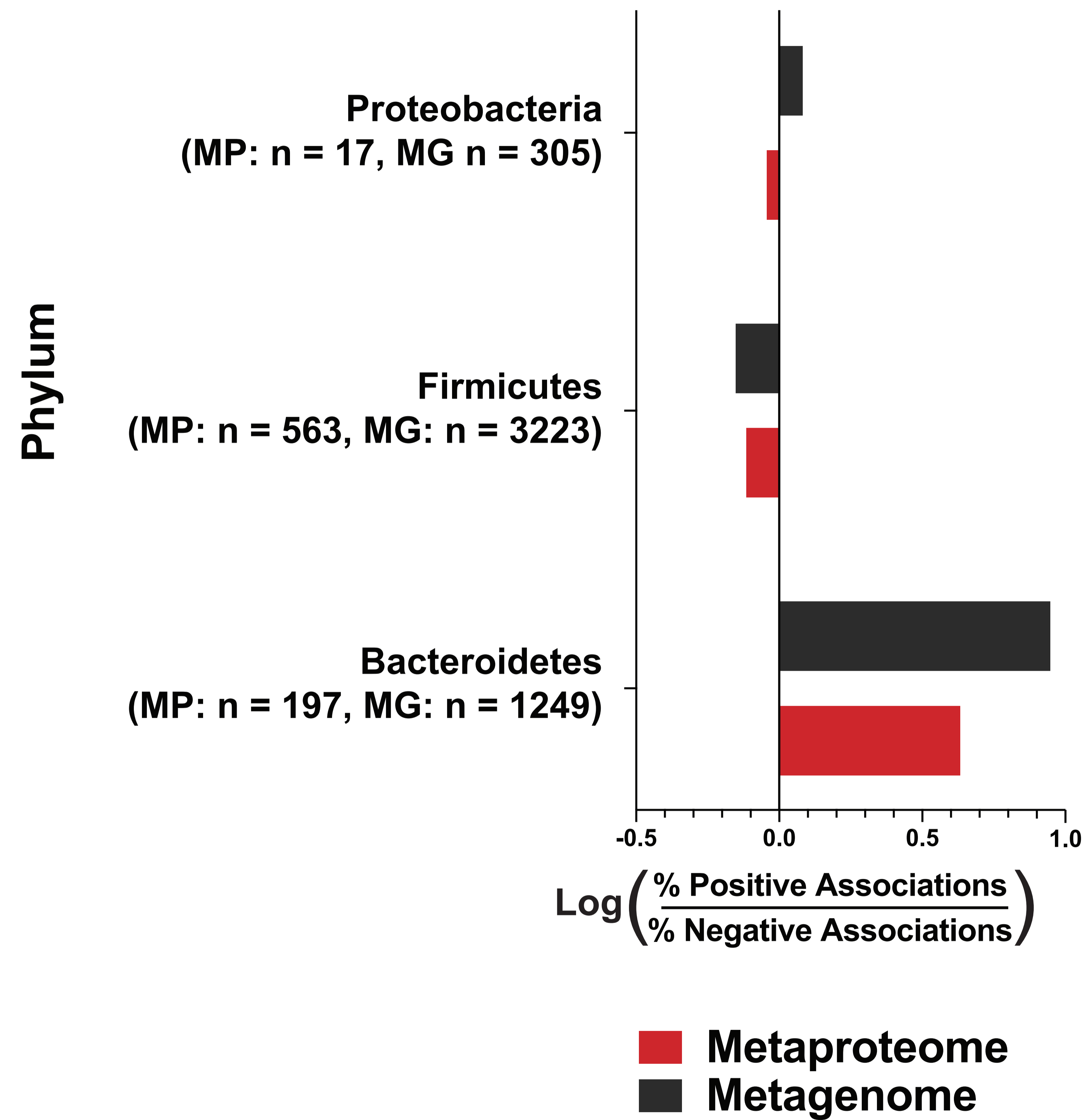**b**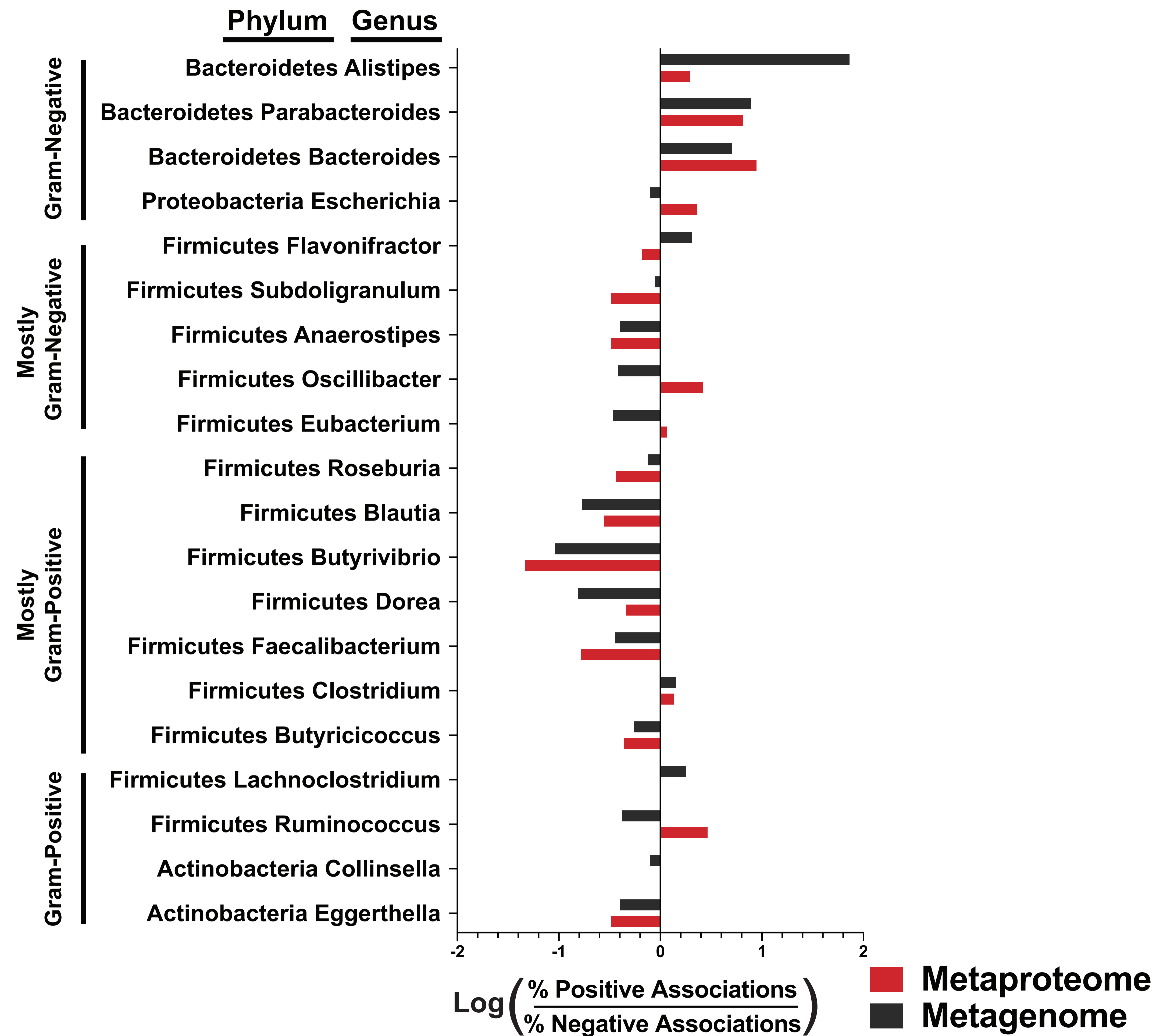

Supplement: FIG S4 [file mSystems.00337-18-sf004.pdf]

a

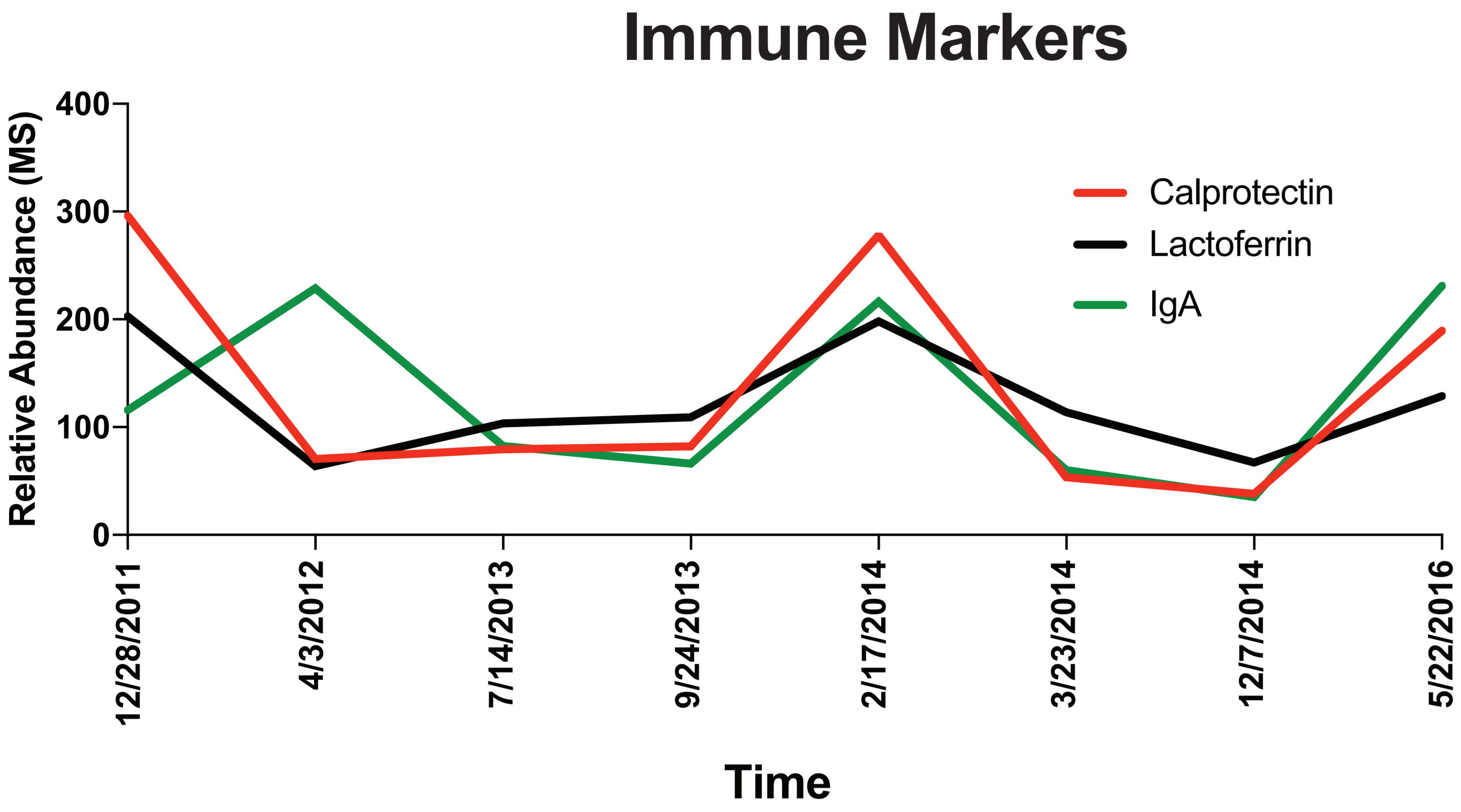

b

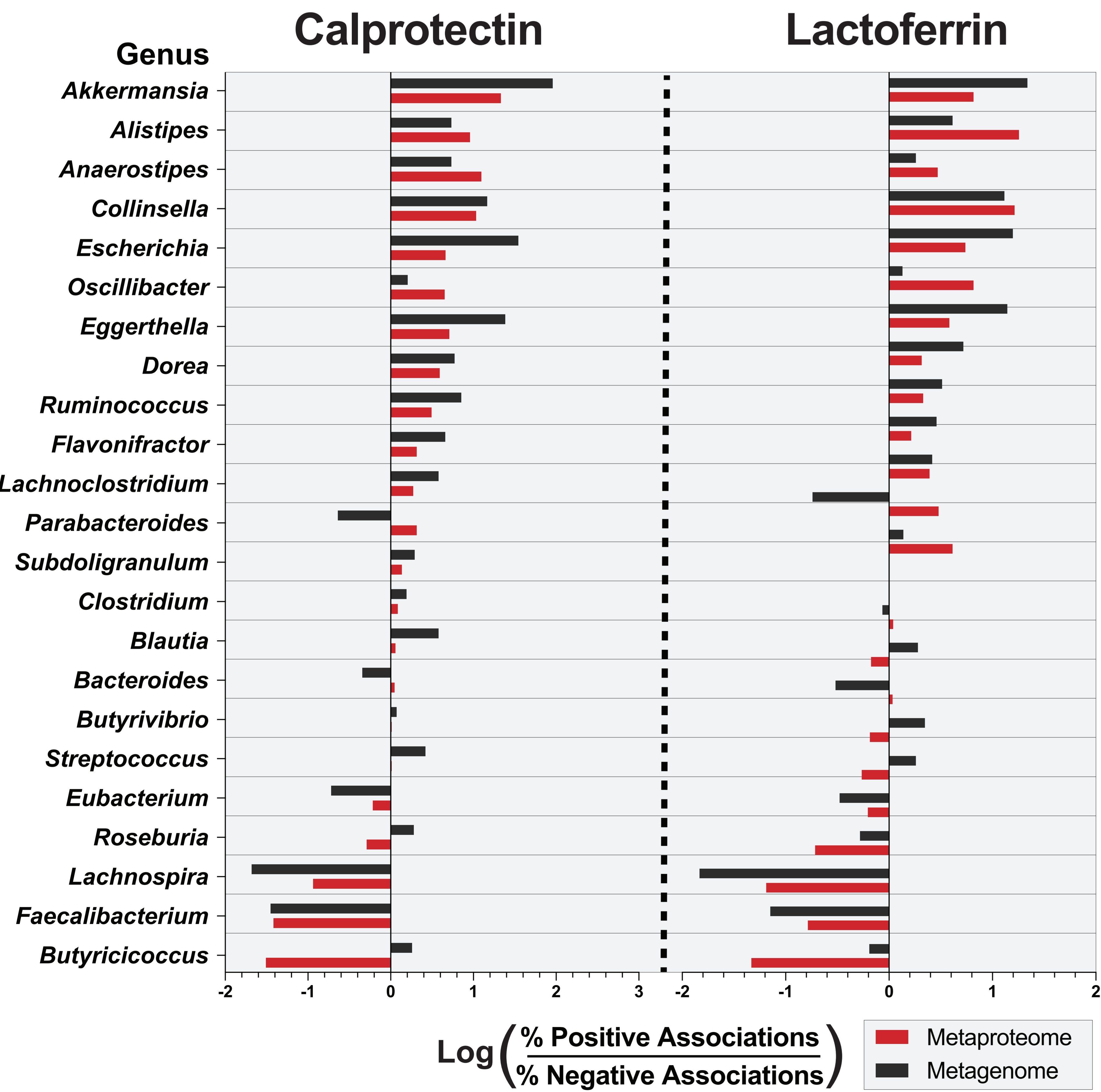

c

eggNOG Category

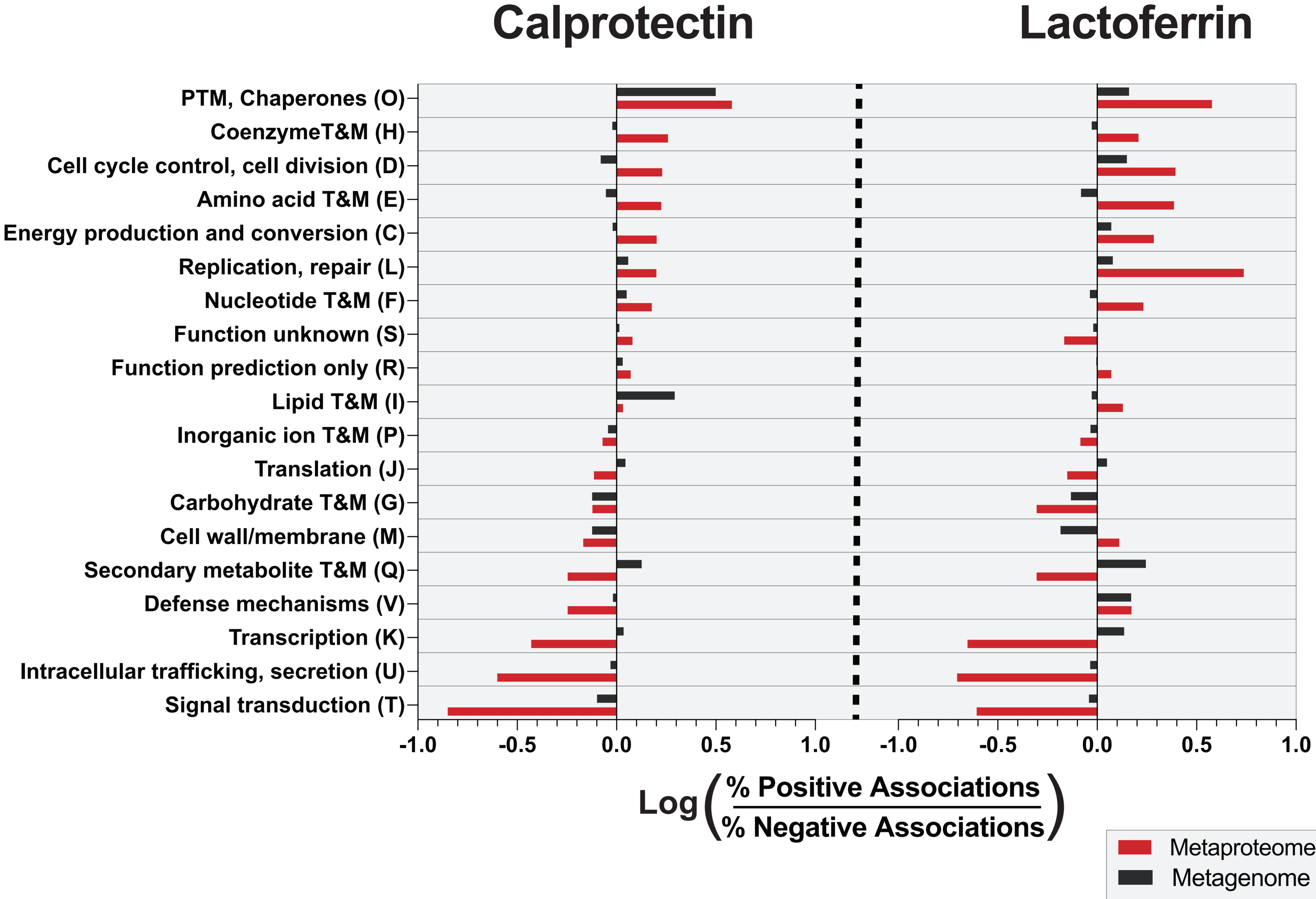

d

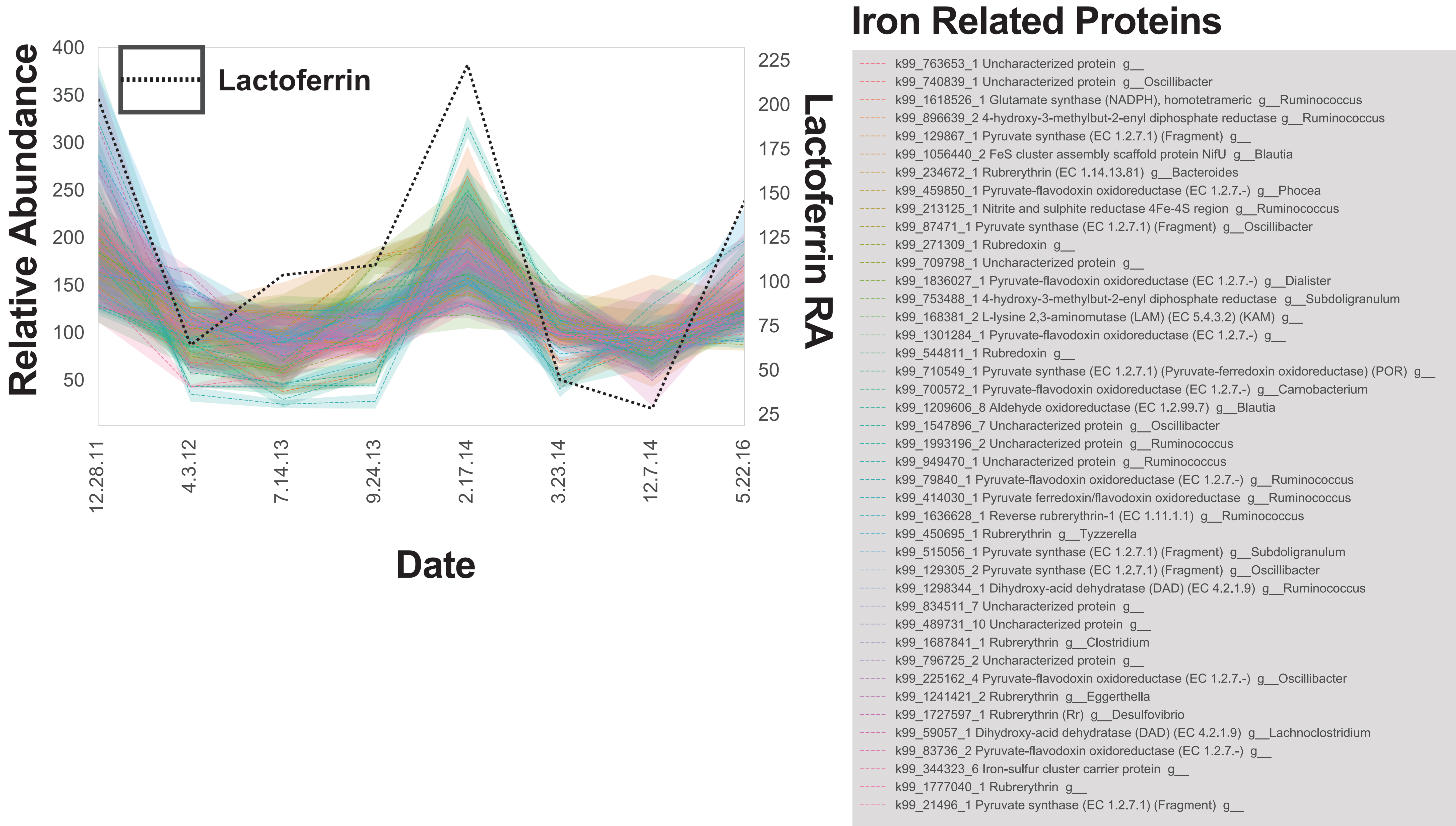

Supplement: FIG S5 [file mSystems.00337-18-sf005.pdf]
