# Supplementary material for: Evaluating Metagenomic Prediction of the Metaproteome in a 4.5-Year Study of a Patient with Crohn's Disease
Source: mSystems. 2019 Feb 12;4(1):e00337-18. doi: 10.1128/mSystems.00337-18 (PMC6372841; doi:10.1128/mSystems.00337-18)
Supplement: TABLE S1 [file mSystems.00337-18-st001.docx]

| **eggNOG Functional Category** | **Mean** | **Median** | **Standard Deviation** | **Count** |
| --- | --- | --- | --- | --- |
| **Energy production and conversion** | 0.28 | 0.29 | 0.42 | 312 |
| **Cell wall/membrane/envelope biogenesis** | 0.39 | 0.45 | 0.36 | 35 |
| **Amino acid transport and metabolism** | 0.35 | 0.38 | 0.40 | 131 |
| **Carbohydrate transport and metabolism** | 0.33 | 0.40 | 0.40 | 340 |
| **Coenzyme transport and metabolism** | 0.27 | 0.35 | 0.46 | 37 |
| **General function prediction only** | 0.29 | 0.32 | 0.42 | 114 |
| **Translation, ribosomal structure and biogenesis** | 0.25 | 0.30 | 0.41 | 382 |
| **Function unknown** | 0.23 | 0.19 | 0.47 | 39 |
| **Nucleotide transport and metabolism** | 0.30 | 0.40 | 0.44 | 77 |
| **Lipid transport and metabolism** | 0.35 | 0.37 | 0.39 | 39 |
| **Transcription** | 0.28 | 0.35 | 0.39 | 68 |
| **Cell cycle control, cell division, chromosome partitioning** | 0.06 | 0.05 | 0.33 | 13 |
| **Intracellular trafficking, secretion, and vesicular transport** | 0.35 | 0.42 | 0.43 | 28 |
| **Replication, recombination and repair** | 0.35 | 0.42 | 0.38 | 32 |
| **Posttranslational modification, protein turnover, chaperones** | 0.32 | 0.44 | 0.41 | 64 |
| **Secondary metabolites biosynthesis, transport and catabolism** | 0.53 | 0.57 | 0.17 | 7 |
| **Inorganic ion transport and metabolism** | 0.39 | 0.54 | 0.44 | 10 |
| **Signal transduction mechanisms** | 0.34 | 0.43 | 0.43 | 12 |
| **Cell motility** | 0.13 | 0.10 | 0.57 | 7 |
| **Defense mechanisms** | 0.34 | 0.55 | 0.46 | 8 |
