# Supplementary material for: Evaluating Metagenomic Prediction of the Metaproteome in a 4.5-Year Study of a Patient with Crohn's Disease
Source: mSystems. 2019 Feb 12;4(1):e00337-18. doi: 10.1128/mSystems.00337-18 (PMC6372841; doi:10.1128/mSystems.00337-18)
Supplement: FIG S2 [file mSystems.00337-18-sf002.pdf]

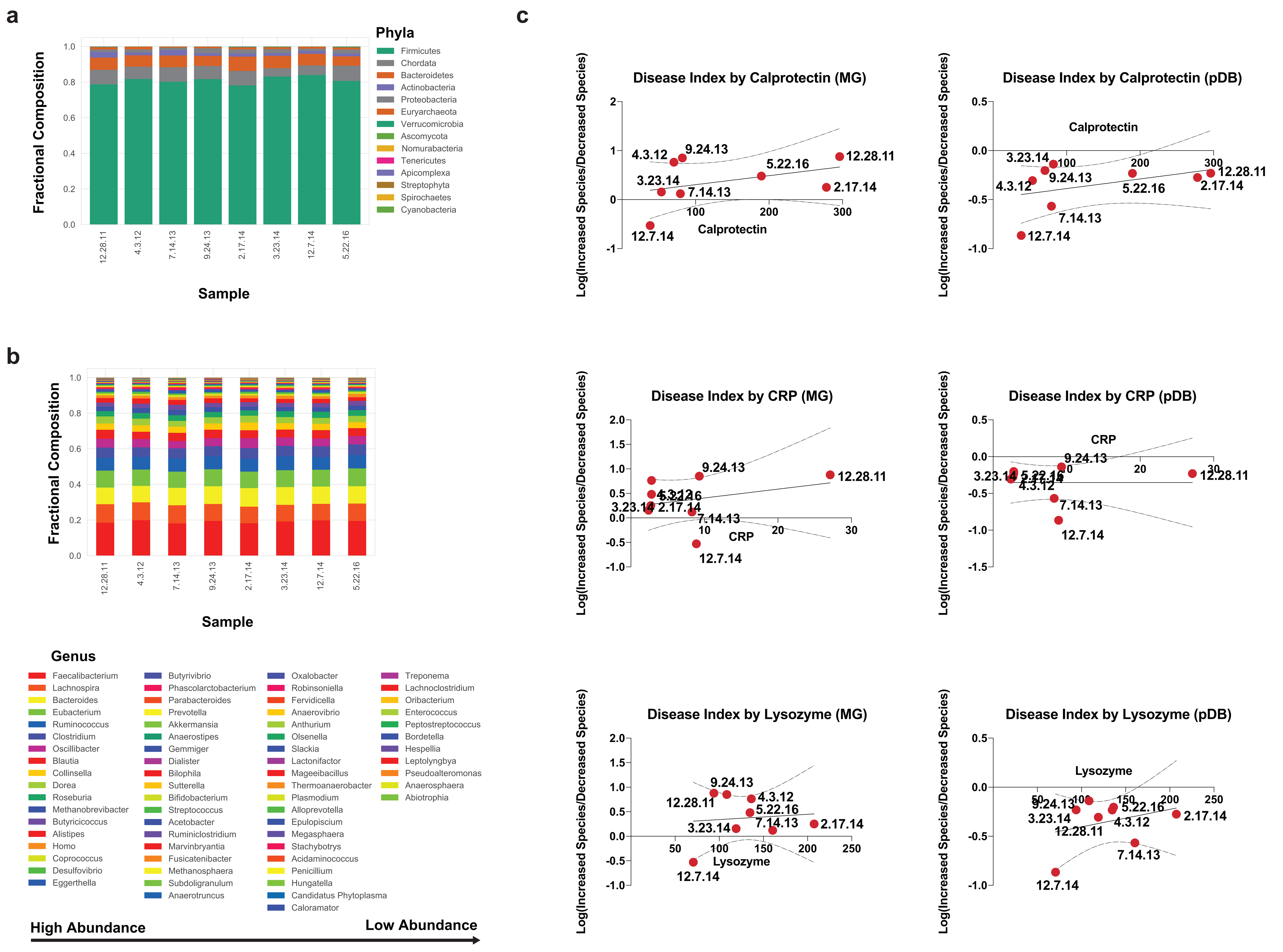
a
Phyla
Firmicutes
Chordata
Bacteroidetes
Actinobacteria
Proteobacteria
Euryarchaeota
Verrucomicrobia
Ascomycota
Nomurabacteria
Tenericutes
Apicomplexa
Streptophyta
Spirochaetes
Cyanobacteria
Fractional Composition
Sample
12.28.11
4.3.12
7.14.13
9.24.13
2.17.14
3.23.14
12.7.14
5.22.16
b
Fractional Composition
Sample
Genus
Faecalibacterium
Lachnospira
Bacteroides
Eubacterium
Ruminococcus
Clostridium
Oscillibacter
Blautia
Collinsella
Dorea
Roseburia
Methanobrevibacter
Butyricicoccus
Alistipes
Homo
Coprococcus
Desulfovibrio
Eggerthella
Butyrivibrio
Phascolarctobacterium
Parabacteroides
Prevotella
Akkermansia
Anaerostipes
Gemmiger
Dialister
Bilophila
Sutterella
Bifidobacterium
Streptococcus
Acetobacter
Ruminiclostridium
Marvinbryantia
Fusicatenibacter
Methanosphaera
Subdoligranulum
Anaerotruncus
Oxalobacter
Robinsoniella
Fervidicella
Anaerovibrio
Anthurium
Olsenella
Slackia
Lactonifactor
Mageeibacillus
Thermoanaerobacter
Plasmodium
Alloprevotella
Epulopiscium
Megasphaera
Stachybotrys
Acidaminococcus
Penicillium
Hungatella
Candidatus Phytoplasma
Caloramator
Treponema
Lachnoclostridium
Oribacterium
Enterococcus
Peptostreptococcus
Bordetella
Hespellia
Leptolyngbya
Pseudoalteromonas
Anaerosphaera
Abiotrophia
High Abundance
Low Abundance
c
Disease Index by Calprotectin (MG)
Disease Index by Calprotectin (pDB)
Calprotectin
Disease Index by CRP (MG)
Disease Index by CRP (pDB)
CRP
Disease Index by Lysozyme (MG)
Disease Index by Lysozyme (pDB)
Lysozyme
Log(Increased Species/Decreased Species)
