# Supplementary material for: Evaluating Metagenomic Prediction of the Metaproteome in a 4.5-Year Study of a Patient with Crohn's Disease
Source: mSystems. 2019 Feb 12;4(1):e00337-18. doi: 10.1128/mSystems.00337-18 (PMC6372841; doi:10.1128/mSystems.00337-18)
Supplement: TABLE S3 [file mSystems.00337-18-st003.docx]

|  | **Calprotectin** | | **CRP** | | **Lysozyme** | |
| --- | --- | --- | --- | --- | --- | --- |
| **Genus** | **pDB** | **MG** | **pDB** | **MG** | **pDB** | **MG** |
| ***Butyricicoccus*** | 87 | 36 | 3 | 104 | 7 | 31 |
| ***Faecalibacterium*** | 187 | 911 | 30 | 301 | 18 | 85 |
| ***Lachnospira*** | 49 | 30 | 78 | 47 | 0 | 0 |
| ***Roseburia*** | 56 | 60 | 64 | 138 | 19 | 100 |
| ***Eubacterium*** | 168 | 309 | 94 | 477 | 64 | 150 |
| ***Streptococcus*** | 2 | 10 | 45 | 126 | 0 | 0 |
| ***Butyrivibrio*** | 18 | 20 | 26 | 49 | 7 | 22 |
| ***Bacteroides*** | 133 | 426 | 63 | 584 | 140 | 473 |
| ***Blautia*** | 277 | 375 | 360 | 1905 | 91 | 148 |
| ***Clostridium*** | 196 | 504 | 133 | 1173 | 57 | 423 |
| ***Subdoligranulum*** | 33 | 107 | 25 | 374 | 8 | 23 |
| ***Parabacteroides*** | 15 | 41 | 23 | 30 | 21 | 59 |
| ***Lachnoclostridium*** | 14 | 112 | 11 | 213 | 4 | 87 |
| ***Flavonifractor*** | 5 | 133 | 3 | 158 | 2 | 82 |
| ***Ruminococcus*** | 255 | 331 | 143 | 1317 | 89 | 151 |
| ***Dorea*** | 69 | 85 | 57 | 396 | 12 | 39 |
| ***Eggerthella*** | 11 | 83 | 46 | 239 | 1 | 12 |
| ***Oscillibacter*** | 137 | 478 | 19 | 186 | 9 | 156 |
| ***Escherichia*** | 20 | 119 | 16 | 244 | 7 | 7 |
| ***Collinsella*** | 44 | 102 | 116 | 410 | 3 | 6 |
| ***Anaerostipes*** | 101 | 39 | 136 | 248 | 2 | 8 |
| ***Alistipes*** | 56 | 78 | 115 | 91 | 6 | 549 |
| ***Akkermansia*** | 43 | 310 | 0 | 0 | 1 | 1 |
| ***Total*** | 1976 | 4699 | 1606 | 8810 | 568 | 2612 |
