# Supplementary material for: Evaluating Metagenomic Prediction of the Metaproteome in a 4.5-Year Study of a Patient with Crohn's Disease
Source: mSystems. 2019 Feb 12;4(1):e00337-18. doi: 10.1128/mSystems.00337-18 (PMC6372841; doi:10.1128/mSystems.00337-18)
Supplement: TABLE S4 [file mSystems.00337-18-st004.docx]

**Supplementary Table 4. Total number of proteins and genes significantly correlated to each eggNOG category.**

|  | **Calprotectin** | | **CRP** | | **Lysozyme** | |
| --- | --- | --- | --- | --- | --- | --- |
| **eggNOG Functional Category** | **pDB** | **MG** | **pDB** | **MG** | **pDB** | **MG** |
| **Amino acid transport and metabolism** | 91 | 209 | 76 | 343 | 22 | 115 |
| **Carbohydrate transport and metabolism** | 166 | 232 | 154 | 467 | 60 | 115 |
| **Cell cycle control, cell division, chromosome partitioning** | 8 | 35 | 10 | 83 | 2 | 13 |
| **Cell wall/membrane/envelope biogenesis** | 22 | 122 | 23 | 240 | 3 | 68 |
| **Coenzyme transport and metabolism** | 21 | 84 | 15 | 172 | 3 | 49 |
| **Defense mechanisms** | 6 | 95 | 8 | 176 | 1 | 40 |
| **Energy production and conversion** | 156 | 193 | 118 | 339 | 47 | 89 |
| **Function unknown** | 25 | 141 | 32 | 302 | 12 | 86 |
| **General function prediction only** | 86 | 275 | 70 | 556 | 20 | 125 |
| **Inorganic ion transport and metabolism** | 10 | 125 | 15 | 202 | 4 | 45 |
| **Intracellular trafficking, secretion, and vesicular transport** | 13 | 62 | 9 | 86 | 3 | 17 |
| **Lipid transport and metabolism** | 29 | 43 | 12 | 89 | 5 | 15 |
| **Nucleotide transport and metabolism** | 62 | 79 | 44 | 147 | 15 | 41 |
| **Posttranslational modification, protein turnover, chaperones** | 54 | 74 | 57 | 160 | 21 | 36 |
| **Replication, recombination and repair** | 19 | 236 | 20 | 400 | 5 | 97 |
| **Secondary metabolites biosynthesis, transport and catabolism** | 2 | 9 | 2 | 11 | 1 | 7 |
| **Signal transduction mechanisms** | 5 | 68 | 9 | 106 | 2 | 30 |
| **Transcription** | 58 | 127 | 18 | 233 | 14 | 49 |
| **Translation, ribosomal structure and biogenesis** | 292 | 254 | 206 | 404 | 72 | 107 |
| **Total** | 1125 | 2463 | 898 | 4516 | 312 | 1144 |
